# New Homogeneous Spatial Areas Identified Using Case-Crossover Spatial Lag Grid Differences between Aerosol Optical Depth-PM_2.5_ and Respiratory-Cardiovascular Emergency Department Visits and Hospitalizations

**DOI:** 10.3390/atmos13050719

**Published:** 2022-04-30

**Authors:** John T. Braggio, Eric S. Hall, Stephanie A. Weber, Amy K. Huff

**Affiliations:** 1 Maryland Department of Health, Baltimore, MD 21201, USA; 2 Mt. Diablo Analytical Solutions and Reporting Institute, LLC (Diablo Analytical Institute, DAI), 3474 Tice Creek Drive, Unit 7, Walnut Creek, CA 94595, USA; 3 U.S. Environmental Protection Agency, Durham, NC 27709, USA; 4 Battelle Memorial Institute, Columbus, OH 43201, USA; 5 Huntington National Bank, Columbus, OH 43215, USA; 6 Battelle Memorial Institute, Arlington, VA 22201, USA; 7 IMSG at NOAA/NESDIS Center for Satellite Applications and Research, 5825 University Research Ct., Suite 3250, College Park, MD 20740, USA

**Keywords:** spatial heterogeneity, AOD-PM_2.5_, respiratory-cardiovascular, lag grids, urban–rural, season

## Abstract

Optimal use of Hierarchical Bayesian Model (HBM)-assembled aerosol optical depth (AOD)-PM_2.5_ fused surfaces in epidemiologic studies requires homogeneous temporal and spatial fused surfaces. No analytical method is available to evaluate spatial heterogeneity. The temporal case-crossover design was modified to assess the spatial association between four experimental AOD-PM_2.5_ fused surfaces and four respiratory–cardiovascular hospital events in 12 km^2^ grids. The maximum number of adjacent lag grids with significant odds ratios (ORs) identified homogeneous spatial areas (HOSAs). The largest HOSA included five grids (lag grids 04; 720 km^2^) and the smallest HOSA contained two grids (lag grids 01; 288 km^2^). Emergency department asthma and inpatient asthma, myocardial infarction, and heart failure ORs were significantly higher in rural grids without air monitors than in urban grids with air monitors at lag grids 0, 1, and 01. Rural grids had higher AOD-PM_2.5_ concentration levels, population density, and poverty percentages than urban grids. Warm season ORs were significantly higher than cold season ORs for all health outcomes at lag grids 0, 1, 01, and 04. The possibility of elevated fine and ultrafine PM and other demographic and environmental risk factors synergistically contributing to elevated respiratory–cardiovascular chronic diseases in persons residing in rural areas was discussed.

## 1. Introduction

### 1.1. Literature Review

Published studies emphasize the detrimental effects of acute and chronic exposure to elevated ambient PM_2.5_ (fine particulate matter) concentration levels on respiratory–cardiovascular chronic disease morbidity [1–18]. Along with PM_2.5_, PM_0.1_ (ultrafine particulate matter) adversely impacts respiratory-cardiovascular chronic diseases [19–26]. A major difference between fine PM and ultrafine PM is aerosol particle size, with the latter being smaller than the former [18,27]. Ambient PM_2.5_ aerosol particles are inhaled through the mouth and nose and travel down the trachea and bronchial tubes deep into the lungs [25,27,28]. Ultrafine PM can traverse lung tissue, enter the circulatory system, and can be deposited into heart tissue [27,29]. Various authors have suggested that PM’s adverse physiologic and health outcome effects are inversely related with aerosol particle size, with smaller aerosol particles producing greater physiologic and epidemiologic disruptions from normal functioning than larger aerosol particles [21,26,28,30,31]. Currently, ongoing population-based PM monitoring only occurs for PM_2.5_, and not for ultrafine PM [20]. Several publications have described associations between ambient ultrafine PM and adverse health outcomes [19,21,23,24,30], but a recent review of epidemiologic studies evaluating the association between PM_0.1_ and respiratory-cardiovascular outcomes concluded that the available results are equivocal [26]. Therefore, at this time, epidemiologic study results have not been able to confirm all the anticipated worse health outcomes following acute or chronic exposure to ultrafine PM compared to acute or chronic exposure to fine PM on respiratory-cardiovascular diseases.

Frequent, accurate, and timely measurements of ambient PM_2.5_ concentration levels, obtained from on-the-ground air monitors, are essential for protecting human health and decreasing respiratory-cardiovascular morbidity and mortality [5,8,11,12,32–35]. There are challenges in using ambient PM_2.5_ air monitors, especially those maintained by the U.S. Environmental Protection Agency Air Quality System in epidemiologic studies [34]. The majority of these PM_2.5_ filter-based monitors measure ambient PM_2.5_ concentration levels every three or six days most often in urban areas with higher population density [34]. Fewer studies have evaluated the effects of ambient fine PM exposure on health outcomes in rural areas [36–42]. Increasing the number of stationary ambient PM_2.5_ air monitors in rural areas would provide accurate readings of fine PM concentration levels and, at the same time, decrease spatial heterogeneity. Epidemiologic studies could use PM_2.5_ readings made in rural areas to determine fine PM’s short- and long-term contribution to the occurrence of respiratory-cardiovascular hospital events [3]. Unfortunately, this type of enhancement to the U.S. Environmental Protection Agency Air Quality System air monitoring network would be expensive to implement and maintain.

An alternative and more cost-effective solution would be to utilize remote sensing methodology instead of expanding the on-the-ground ambient fine PM monitoring network, especially in rural areas [3,6,7,15,17,36,40,42–45]. Different investigators have combined satellite aerosol optical depth (AOD) readings with PM_2.5_ air monitor measurements by utilizing HBM or other statistical procedures to attain continuous (homogeneous) temporal-spatial aerosol concentration level fused surfaces that purport to accurately represent ambient PM_2.5_ measurements in urban areas that have air monitors as well as concentration level estimates in rural areas that do not have air monitors [3,15,42,46–50]. Urban and rural areas also differ on residents’ demographics. Urban areas have more poverty, greater population density, and more non-Hispanic non-White residents than rural areas [3,33,37,38]. Numerous publications have emphasized the importance of describing the temporal and spatial attributes of PM_2.5_ concentration levels measured by ambient air monitors [33,51–54] and AOD-PM_2.5_ model estimates in areas with and without air monitors [3,42,47,55–61].

### 1.2. Research Questions

The 99 12 km^2^ CMAQ (Community Multiscale Air Quality) grids that define the Baltimore study area are temporally and spatially heterogeneous on resident demographics, placement of on-the-ground air monitors, and ambient PM_2.5_ concentration levels. However, the Baltimore study area’s spatial heterogeneity consists of differences in ambient fine PM concentration levels between urban grids with air monitors and rural grids without air monitors [3]. Urban grids with air monitors should represent a more spatially homogeneous area given the *a priori* criteria that were used to select those locations to install permanent on-the-ground ambient air monitors. The 15 urban grids with air monitors in the Baltimore study area appear to share similar demographic and air pollution attributes that include higher poverty, greater population density, and elevated ambient PM_2.5_ concentration levels. Since the number of rural grids without air monitors is higher than the number of urban grids with air monitors by 560%, it is possible that there could be a smaller and more homogeneous subgroup of rural grids without air monitors that resembles the urban grids with air monitors by also demonstrating higher poverty, population density, and elevated ambient PM_2.5_ concentration levels. Residents in higher risk homogeneous rural grids should also resemble residents in urban grids by displaying a similar association between elevated fine PM concentration levels and increases in respiratory-cardiovascular emergency department (ED) visits and inpatient (IP) hospitalizations.

To identify homogeneous spatial areas (HOSAs) that demonstrate a similar relationship between elevated AOD-PM_2.5_ concentration levels and increased respiratory-cardiovascular ED visits or IP hospitalizations, we modified, for the first time, the temporal lag day case-crossover design to identify homogeneous spatial grids. In the temporal lag day case-crossover design, all exposure-health outcome assessments occur in the patients’ grid of residence. The date when the patient received medical care is the index day or lag day 0. The day preceding the index day is lag day 1. The two to four days before the index day are identified as lag days 2–4. The spatial lag grid case-crossover design evaluates the association between exposure and outcome on the same day but in different grids. The patients’ grid of residence is the index grid. It has a lag grid value of 0. The grid that is next to and spatially precedes the index grid is lag grid 1. The two to four grids that spatially precede the index grid are referred to as lag grids 2–4. The lag day and lag grid case-crossover formatted linked exposure–health outcome data files were both analyzed using conditional logistic regression (CLR). CLR analyses compute odds ratios (ORs) for lag days and lag grids. Significant ORs identify lag days or lag grids with elevated AOD-PM_2.5_ concentration levels and increased respiratory-cardiovascular ED visits or IP hospitalizations.

HOSAs represent grids with significant ORs, while heterogeneous spatial areas included all other grids that do not have significant ORs. HOSA size in km is determined by the number of adjacent grids that have significant ORs and the total area in km^2^. Since each HOSA consists of a single CMAQ grid with the dimensions of 12 km wide by 12 km tall, or 144 km^2^, the size of the HOSA will be a multiple of 144 km^2^. By implementing the spatial lag grid case-crossover design in the Baltimore study area, it should be possible to identify HOSAs that share the same relationship between AOD-PM_2.5_ and PMB fused surface concentration levels and increased respiratory-cardiovascular ED visits or IP hospitalizations in all grids, in urban grids with air monitors, and in rural grids without air monitors. To our knowledge, this novel use of the traditional temporal lag day case-crossover design to identify spatial lags grids and the maximum length of similar interconnected lag grids that operationally define HOSAs has not been reported in the published scientific literature.

Differences between warm and cold seasons were evaluated in the New York City [17] and Baltimore [3] study areas. Significantly higher ORs were found during the warm season versus the cold season in the Baltimore study area, but not in the New York City study area. Similar warm and cold season analyses are conducted in this spatial lag grid case-crossover data analysis study, as they were previously implemented utilizing the temporal lag day case-crossover design in the New York City and Baltimore study areas.

### 1.3. Research Objectives

The first objective of this research is to determine the size of HOSAs in the entire Baltimore study area and in urban grids with air monitors and in rural grids without air monitors. The second aim is to evaluate AOD-PM_2.5_ and baseline PMB fused surface concentration levels in urban grids with air monitors and in rural grids without air monitors. The third goal is to assess warm and cold season differences in in the entire Baltimore study area. The final objective is to review the results from this spatial lag grid case-crossover study and the previously completed temporal lag day case-crossover study [3] to identify similarities and differences between the spatial lag grid analysis and the temporal lag day analysis.

## 2. Materials and Methods

We implemented the spatial lag grid case-crossover design and utilized the same four experimental AOD-PM_2.5_ and PMB concentration level fused surfaces and the same four respiratory-cardiovascular chronic disease ED visits and IP hospitalizations that were previously used in the temporal lag day case-crossover studies completed in the Baltimore [3] and New York City [17] study areas. This section includes spatial lag grid case-crossover study details. Other specific information about the temporal lag day analyses is available in earlier publications describing the Baltimore [3] and New York City [17] study areas.

### 2.1. Baltimore Study Area

**Figure 1.**
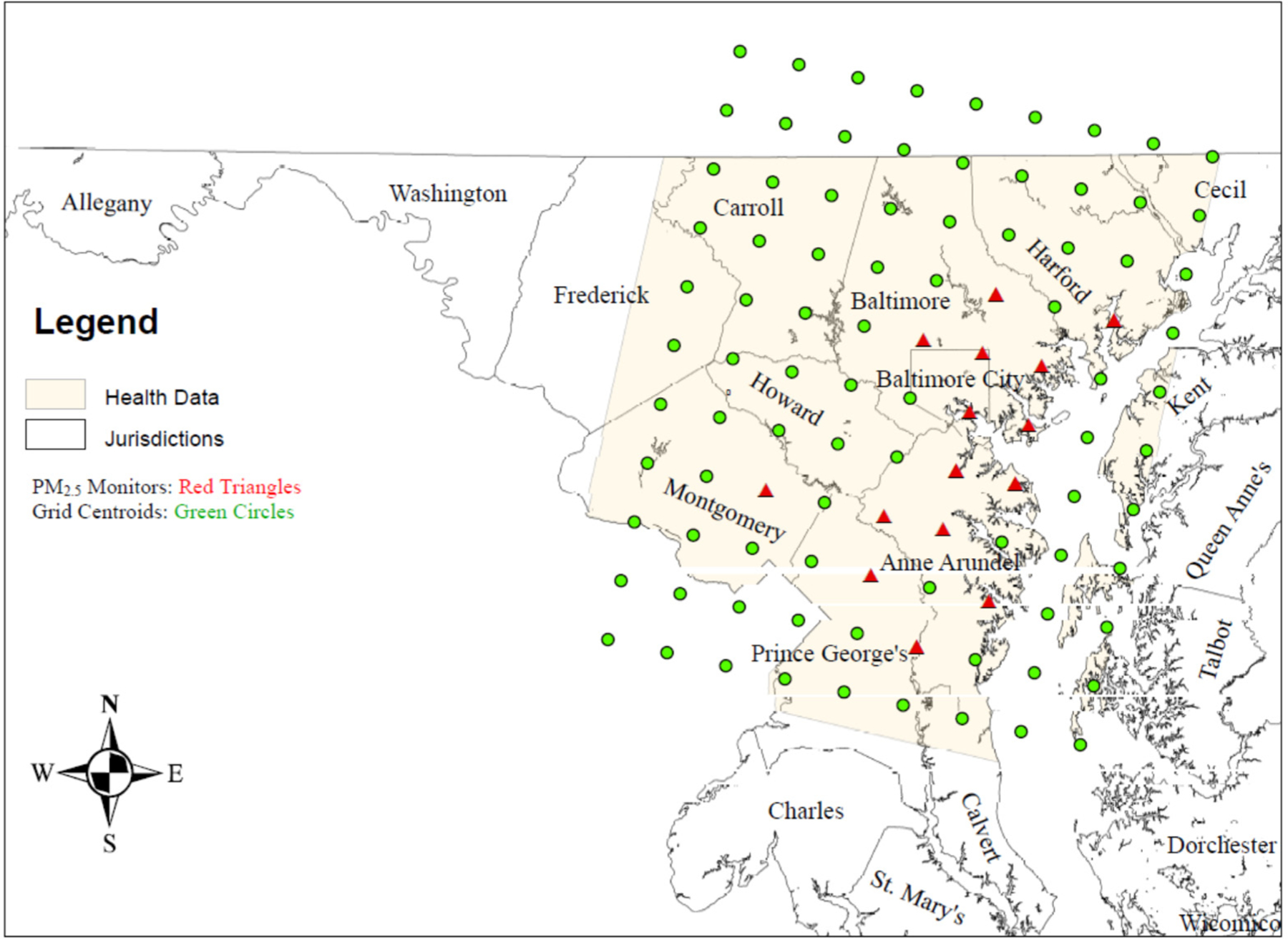
Maryland choropleth map of the Baltimore study area. Map from Reference [80].

Figure 1 is a map of the Baltimore study area. Map layers include (1) jurisdiction names (counties and Baltimore city); (2) centroids (green circles) for the 11 (south [S] to north [N]) by 9 (west [W] to east [E]) 99 12 km^2^ CMAQ grids—these are the grids that define the Baltimore study area; (3) location of the 17 federal reference method ambient PM_2.5_ air monitors (red triangles); and the (4) availability of respiratory–cardiovascular chronic disease hospitalization data.

Table 1 provides additional specific location information about the 99 CMAQ grids in terms of the 17 federal reference method PM_2.5_ air monitors, which were operational in 2004–2006, in the 15 urban grids with air monitors. The grids with ambient air monitors are identified with row-column grid coordinates, and city/county abbreviations, and are displayed in Table 1. There were 6 air monitors in Baltimore city, 4 in Anne Arundel, 3 in Prince George’s, 2 in Baltimore, and 1 each in Montgomery county and Hartford county.

#### 2.1.1. ED and IP Hospitalization Cases

The assessment included the four respiratory-cardiovascular chronic disease hospitalization end points, consisting of ED visits (asthma) and IP hospitalizations (asthma, myocardial infarction, MI; heart failure, HF). The 4 health data files contained all the 2004–2006 ED visits and IP hospitalizations in Maryland reported by State statute to the Maryland Health Services Cost Review Commission [62]. Individual electronic patient records did not include patient names, social security numbers, or residential addresses. However, each electronic patient record had temporal information about the ED visits and IP hospitalizations: year (2004–2006), quarter (winter, spring, summer, fall), day of week (Sunday through Saturday), date of birth, gender, race, health insurance, spatial location for the residential address, five-digit U.S. Postal Service residential Zone Improvement Plan (ZIP) code [63], and one primary and multiple secondary diagnostic fields, with International Classification of Diseases, Ninth Revision, Clinical Modification (ICD-9-CM) billing codes [64]. ICD-9-CM codes permitted the identification of electronic patient records with ED asthma visits and IP asthma (493), MI (410), and HF (428) hospitalizations in the primary diagnosis field. In addition, ICD-9-CM codes identified which electronic patient records had one or more of the three comorbid chronic diseases of atherosclerosis (414, 440), diabetes mellitus (250), and hypertension (401). The study’s protocol, including the proposed data analyses, were approved by the Maryland Department of Health Institutional Review Board [65] and the Maryland Health Services Cost Review Commission [62]. The Maryland Department of Health’s Institutional Review Board concluded that since this study only utilized administrative hospital data, and because there was no contact with patients, it was approved through an exempted review.

#### 2.1.2. Controls

There were three controls for each case. While the electronic patient records for the controls were the same as those for the cases, the cases had different quarterly PM_2.5_ exposure values than the quarterly PM_2.5_ exposure values assigned to the controls. Three different monthly control exposure sampling strategies were utilized: the first control was assigned the mean of the PM_2.5_ concentration levels for January (winter), April (spring), July (summer), and October (fall); the second control included mean PM_2.5_ concentration levels for February (winter), May (spring), August (summer), and November (fall); and the third control comprised mean PM_2.5_ concentration levels for March (spring), June (summer), September (fall), and December (winter).

#### 2.1.3. Case and Control Strata

Each stratum included one case and three controls. There were four different quarterly strata, each including a case with a different quarterly mean PM_2.5_ concentration level exposure value. Three controls were in each of the four quarterly strata but, as stated above, they differed from the cases on the assigned mean quarterly PM_2.5_ concentration level values. The case and control means of the monthly ranks were used to confirm that the Braggio et al. [3] study utilized a bidirectional temporal lag day case-crossover design [66]. Months were ordered in ascending order, from January (1) through December (12), and then the 1–12 monthly ordinal ranks were assigned to each month. Cases with quarterly exposure values had mean monthly ranks of 2.0 for winter, 5.0 for spring, 8.0 for summer, and 11.0 for fall. The mean monthly ranks for the three controls were 5.5 for the first control (1 = January; 4 = April; 7 = July; 10 = October), 6.5 for the second (2 = February; 5 = May; 8 = August; 11 = November), and 7.5 for the third (3 = March; 6 = June; 9 = September; 12 = December). For the first and second strata, mean quarterly ranks for the three controls were higher than the mean quarterly ranks for the two cases. In the last two strata, the mean quarterly ranks for the three controls were lower than the mean quarterly ranks for the two cases. Each case and the three associated controls were matched on age, gender, race, health insurance, residential ZIP code, year, and day of the week. PM_2.5_ concentration levels and effect modifiers varied temporally by year (3), quarter (4), day of the week (7), and spatially by CMAQ grid (99), thereby resulting in 8316 different possible variable combinations for the four AOD-PM_2.5_ and PMB fused surfaces. Of the 99 CMAQ grids with complete AOD-PM_2.5_ and baseline PMB fused surface fine PM concentration level values, 72 grids also had associated health data: all 15 grids with air monitors, and 57 of the 84 without air monitors. Warm and cold season differences were preserved for subsequent analyses by using this bidirectional lag grid case-crossover design.

### 2.2. Confounders and Effect Modifiers

The comorbid health conditions included atherosclerosis, hypertension, and diabetes. Other confounders were pollen and apparent temperature. Apparent temperature includes ambient temperature (*°*F), wind speed, and relative humidity [3,17]. Ambient temperature values were computed for grids stratified on year, month, and day. Major holidays (and the day after) were coded as dummy variables (1 = holiday or the day after, 0 = no holiday) in each annual ED visit and IP hospitalization file. Dummy variables were used to code snowstorms (1 = yes, 0 = no) in each of the three annual files.

Poverty and population density were two geographic-based demographic variables that came from the U.S. Census Bureau [67] and the Maryland Department of Planning, Maryland Data Center [68]. Maryland Zip Code Tabulation Area (ZCTA) spatial polygons for poverty and persons per square mile were obtained from the U.S. Census Bureau website [67].

### 2.3. AOD-PM_2.5_ and Baseline PMB Fused Surfaces

The same four experimental AOD-PM_2.5_ and baseline PMB concentration level fused surfaces, previously described in detail in earlier publications [3,17], were used in this spatial lag grid case-crossover data analysis study. Two orbiting satellites, Terra and Aqua, obtained daily readings, the first one in the morning (10:30 a.m.) and the other in the afternoon (1:30 p.m.), local time. The AOD measurement column was continuous from each satellite’s trajectory in space to the Earth’s surface. PM_2.5_ particles within the AOD column changed the light refraction properties, thereby making it possible to obtain AOD unitless measurements. Thus, the AOD readings represented proxies for ambient PM_2.5_ measurements. The Moderate Resolution Imaging Spectroradiometer Collection 5 AOD data files for 2004–2006, at a 10 km^2^ resolution, were downloaded from the National Aeronautics and Space Administration Level 1 and Atmosphere Archive and Distribution System. The Moderate Resolution Imaging Spectroradiometer data were remapped to CMAQ’s 12 km^2^ grid system. The previously established relationship between the AOD unitless values and the ambient PM_2.5_ measurements was used to assign a corresponding PM_2.5_ concentration level value to each AOD unitless value. Implementation of this algorithm resulted in a continuous space-time AOD-PM_2.5_ fused surface [69–71].

The updated HBM permitted the fusing of two or three different input surfaces with (not-Kriged) or without (Kriged) missing AOD-PM_2.5_ concentration level values. **PMC** represented the inclusion of ambient PM_2.5_ monitor measurements with AOD-PM_2.5_ concentration levels. Satellite recording failure or the presence of cloud cover interfered with obtaining the satellite’s recording of unitless AOD column readings, thereby resulting in missing data values in the PMC fused surface. To minimize the loss of daily AOD readings, a second AOD-PM_2.5_ fused surface was Kriged, resulting in the **PMCK** fused surface—the HBM fusion of monitor PM_2.5_ measurements with Kriged PMC concentration levels. For the two remaining AOD-PM_2.5_ fused surfaces, the HBM was used to combine PMC (not Kriged) or PMCK (Kriged) with monitor PM_2.5_ measurements and CMAQ PM_2.5_ model estimates to produce **PMCQ** and **PMCKQ**, respectively. Using the HBM, it was also possible to assemble the updated **PMB**—the baseline fused surface—by combining ambient PM_2.5_ monitor measurements with CMAQ PM_2.5_ model estimates. The four experimental AOD-PM_2.5_ and baseline PMB fused surfaces were displayed within the CMAQ grid system by overlying the National Aeronautics and Space Administration native 10 km^2^ AOD grid onto the U.S. Environmental Protection Agency native 12 km^2^ CMAQ grid.

Table 2 summarizes how the HBM was used to statistically fuse the four input surfaces (first column) to form the four AOD–PM_2.5_ and baseline PMB fused surfaces (remaining columns). The starting point for all five fused surfaces was the inclusion of the monitor surface, as shown in the first column of Table 2. The monitor surface included ambient PM_2.5_ monitor recordings.

The first three fused surfaces, columns 2 through 4 (baseline PMB, PMCQ, and PMCKQ), had the CMAQ PM_2.5_ model estimates input surface. The last two rows and the two middle two columns in Table 2 clearly show differences between PMCQ and PMCKQ—the former included AOD-PM_2.5_ with missing readings and the latter had the AOD_K-_PM_2.5_ Kriged surface without missing readings. Likewise, the last two rows and the last two columns in Table 2 display how the other two AOD-PM_2.5_ fused surfaces were assembled: AOD-PM_2.5_ with missing readings was added to ambient monitor PM_2.5_ recordings to form PMC, and AOD_K-_PM_2.5_ without missing readings was combined with monitor PM_2.5_ recordings to form PMCK. Based on how the four AOD-PM_2.5_ fused surfaces were assembled, one prediction could be that PMCQ and PMCKQ should more accurately align with ambient PM_2.5_ recordings than PMC and PMCK, because only the first two AOD-PM_2.5_ fused surfaces had two different input surfaces that provided continuous (and uninterrupted) spatial coverage in grids with and without air monitors.

### 2.4. Baseline PMB and AOD-PM_2.5_ Correlations

Correlations between the four AOD-PM_2.5_ fused surfaces and baseline PMB were computed for the entire Baltimore study area, and in grids with and without air monitors. In grids with air monitors, PMB concentration values are influenced more by the presence of air monitors and less by CMAQ PM_2.5_ model estimates. In grids without air monitors, PMB concentration level values reflect the greater influence of CMAQ PM_2.5_ model estimates. Correlations between PMB and the four AOD-PM_2.5_ fused surfaces should be higher in grids with air monitors than in grids without air monitors. This outcome should confirm that the concentration levels of the five fused surfaces are influenced by the addition of ambient fine PM measurements. Lower correlations in grids without monitors should represent the differential contribution of AOD-PM_2.5_ with or without CMAQ PM_2.5_ model estimates to each of the four experimental AOD-PM_2.5_ fused surfaces.

### 2.5. File Linkage

The use of a previously developed polygon correspondence file minimized the spatial mismatch between the various irregularly shaped polygons [72]. The polygon correspondence file made it possible to assign each U.S. Postal Service ZIP code polygon (health outcomes), U.S. Census Bureau ZCTA polygon (poverty percent and population density), and CMAQ 12 km^2^ grid template (four experimental AOD-PM_2.5_ and PMB concentration level fused surfaces) to only one CMAQ grid. Assembling the polygon correspondence file necessitated completing the following steps. (1) Obtain latitude-longitude centroid coordinates for each ZIP code and ZCTA polygons [67,68]. (2) Use a geographic information system to assign each residential ZIP code polygon or ZCTA polygon to a single CMAQ 12 km^2^ grid, based on the spatial location of each latitude-longitude centroid coordinate of each polygon within a specific CMAQ grid [73,74]. By using the correspondence file, it was possible to implement the CMAQ 12 km^2^ grid system as the standardized spatial polygon template for this data analysis study. Each of the four linked AOD-PM_2.5_ files (one for ED asthma, a second for IP asthma, a third for MI, and a fourth for HF) was sorted on columns first, and then sorted on rows second.

### 2.6. Spatial Lag Grid and Temporal Lag Day Analyses

The spatial lag grid case-crossover analysis resembled the temporal lag day case-crossover analysis, except the spatial analysis evaluated lag grids, including the index grid, which always had a value of 0 (as well as all lag grids 0–4, 01, 24, and 04), while the temporal analysis assessed lag days preceding the index day, which always had a value of 0 (in addition to all lag days 0–4, 01, 24 and 04). In the New York City and Baltimore publications, we utilized a bidirectional lag day case-crossover design to determine if there were differences due to asthma ED visits and IP asthma, MI, and HF hospitalizations among the individual lag days (0–4) and summary lag days (01, 24, and 04). The lag day analysis determined if ambient PM_2.5_ concentration levels, evaluated by the four experimental AOD-PM_2.5_ and baseline PMB fused surfaces, differentially contributed to the subsequent occurrence of one or more respiratory-cardiovascular ED visits or IP hospitalizations. As stated previously, in the lag day analysis, all outcomes were evaluated on different days but in the same grid. In the lag grid analysis, all outcomes were evaluated in different grids but on the same day.

The primary objective of the lag grid case-crossover assessment was to identify the number of 12-km-wide grids from the index grid that demonstrated the same association between experimental AOD-PM_2.5_ or baseline PMB fused surface concentration levels and one of the four respiratory–cardiovascular ED visits or IP hospitalizations—homogeneous spatial areas (HOSAs).

The implementation of the spatial lag grid case-crossover analysis is described in the three examples shown in Table 3. The first illustration is for grids without air monitors (top of the table). The Spatial Lag Grid Analyses columns display lag grid values, both individual (0–4) and summary measures (01, 24, 04). The grid location for the individual grids with monitors is shown in bold red font for grids with air monitors and bold black font for grids without air monitors. The index grid location for the no monitor example is Row = 9 and Column = 7 (R_9_, C_7_). This index grid has a lag grid value of 0. Moving from the far-right side of the table (E) to the left side of the table (W), the grid adjacent to and immediately to the left of the index grid has a lag grid value of 1, and its grid location is R_9,_ C_6_. The next three lag grids, with lag grid values 2–4, have sequential grid locations: R_9,_ C_5_; R_9,_ C_4_; and R_9,_ C_3_. Summary lag grids 01, 24, and 04 were obtained by computing means for individual lag grid values. To illustrate, the summary lag grid 01 represents the mean for lag grids 0 and 1. Lag grid 24 includes the mean for lag grids 2–4. Lag grid 04 refers to the mean for lag grids 0–4.

The second example, shown in the middle of the table, is for lag grids with air monitors. Here, the index grid location is R_6_, C_7_. Again, the index grid has a lag grid value of 0. The first two lag grids with monitors are in the 6th row, while the next three lag grids with monitors are in the 7th row. Notice that column location decreases by 1 as the lag grid value increases by 1.

The third example, at the bottom of Table 3, is for both monitor grid conditions combined. The index grid location is R_4_, C_7_. All five individual lag grids, with lag grid values of 0–4, are in the 4th row. Three of the five lag grids, 1, 2, and 4, include air monitors. The other two lag grids, 0, and 3, represent grids without monitors.

Considering the spatial lag grid analysis descriptions summarized in Tables 1 and 3 above, it is possible to demonstrate that the three types of lag grid analyses described in Table 3 represent the inclusion of lag grids that either preceded or followed the index grid, based on the index grid column location. Each complete lag grid sequence always includes five individual lag grid values, the index grid, and four additional lag grids. The four lag grids are selected from 1–4 lag grids that always precede the index grid. This prior selection of lag grids, relative to the location of the index grid, occurs in columns 9 E to 5 W. For the remaining four columns, 4 E to 1 W, the selection of the four preceding lag grids, relative to the location of the index grid, will include 1–4 grids that always follow the index grid. To illustrate, when the index grid has a column location value of 4, the selection of the four preceding grids will result in the inclusion of column 3 for lag grid 1, column 2 for lag grid 2, column 1 for lag grid 3, and column 9, in one row above, for lag grid 4. When the index grid is in column 3, two grids precede the index grid, and two grids follow the index grid. The index grid for column 2 includes 1 grid that precedes the index grid and three grids that follow the index grid. For the 4^th^ lag grid, when the index grid is in column 1, all four lag grids follow the index grid and are in the above row.

The second example, shown in the middle of the table, is for lag grids with air monitors. Here, the index grid location is R_6_, C_7_. Again, the index grid has a lag grid value of 0. The first two lag grids with monitors are in the 6th row, while the next three lag grids with monitors are in the 7th row. Notice that column location decreases by 1 as the lag grid value increases by 1.

The third example, at the bottom of Table 3, is for both monitor grid conditions combined. The index grid location is R_4_, C_7_. All five individual lag grids, with lag grid values of 0–4, are in the 4th row. Three of the five lag grids, 1, 2, and 4, include air monitors. The other two lag grids, 0, and 3, represent grids without monitors.

Considering the spatial lag grid analysis descriptions summarized in Tables 1 and 3 above, it is possible to demonstrate that the three types of lag grid analyses described in Table 3 represent the inclusion of lag grids that either preceded or followed the index grid, based on the index grid column location. Each complete lag grid sequence always includes five individual lag grid values, the index grid, and four additional lag grids. The four lag grids are selected from 1–4 lag grids that always precede the index grid. This prior selection of lag grids, relative to the location of the index grid, occurs in columns 9 E to 5 W. For the remaining four columns, 4 E to 1 W, the selection of the four preceding lag grids, relative to the location of the index grid, will include 1–4 grids that always follow the index grid. To illustrate, when the index grid has a column location value of 4, the selection of the four preceding grids will result in the inclusion of column 3 for lag grid 1, column 2 for lag grid 2, column 1 for lag grid 3, and column 9, in one row above, for lag grid 4. When the index grid is in column 3, two grids precede the index grid, and two grids follow the index grid. The index grid for column 2 includes 1 grid that precedes the index grid and three grids that follow the index grid. For the 4th lag grid, when the index grid is in column 1, all four lag grids follow the index grid and are in the above row.

These are the results for all grids that preceded or followed the index grid: For grids with monitors, 62.0% of the grids preceded the index grids, and 38% followed the index grids. For grids without monitors, 69.3% preceded the index grids, and 30.7% followed the index grids. The combined monitor and no monitor condition included 68.4% of the grids that preceded the index grids, and 31.6% of the grids that followed the index grids. To summarize, these results support the conclusion that this spatial lag grid case-crossover design had lag grids that preceded or followed the index grid. This outcome demonstrates that we implemented a bidirectional spatial lag grid case-crossover design [3,66].

### 2.7. Statistical Analyses

The 20 linked exposure-health outcome files (four experimental AOD-PM_2.5_ and baseline PMB fused surfaces with ED asthma, IP asthma, MI, and HF) were analyzed using conditional logistic regression (CLR) statistical software included in the SAS (Statistical Analysis System)/STAT proportional hazards regression (PHREG) Procedure, version 14.3, along with Base SAS version 9.4 [75–79]. PHREG performs CLR analyses on survival data, based on the Cox Proportional Hazards model, by quantifying the effects of explanatory variables on survival times. The chi-square test evaluated group differences between categorical variables, such as case and control status, and race [77]. Assessment of continuous variables of poverty percent and population density (converted to L_10_ values before analysis) involved comparing a case group mean to a control group mean and its associated 95% confidence interval (CI). Means and 95% CIs were computed by Proc Means in Base SAS [77]. If the case mean was below or above the 95% CI lower or upper limit of the control group mean, this outcome was significantly different at *p* ≤ 0.05 [75]. If the case mean was within the 95% CI lower and upper limits of the control mean, this outcome was not significant, *p* > 0.05.

#### 2.7.1. Variable Selection

The CLR runs consisted of a PM_2.5_ fused surface (baseline PMB, PMC, PMCK, PMCQ, PMCKQ) and one respiratory-cardiovascular endpoint (ED asthma, IP asthma, MI, or HF). CLR runs evaluated individual lag grids 0–4, and summary lag grids 01, 24, and 04. The base CLR analysis controlled for the following confounders: apparent temperature, AT—at each lag grid; AT^2^—at lag grids 0, 1, 01, and 04; pollen; snowstorms; and major holidays. Four separate CLR runs evaluated effect modifiers: (1) diabetes mellitus, hypertension, and atherosclerosis; (2) gender, age, and race; (3) health insurance coverage, poverty percent, and population density; and (4) season. An effect modifier was included in subsequent analyses if the initial CLR OR had a probability value of *p ≤* 0.09.

#### 2.7.2. Final CLR Runs

Final CLR runs included the base model, effect modifiers for lag grids 0–4, and summary lag grids 01, 24, and 04. The three monitor grid conditions included grids without a monitor (No), grids with at least one monitor (Yes), and grids with or without air monitors (Both). The null hypothesis was rejected if *p* ≤ 0.05. The use of the Akaike information criterion permitted the selection of CLR outcomes with lower values, thereby representing a better parameter fit [75,76,78]. Follow-up analyses evaluated the contribution of monitor (No, Yes) and season (Cold, Warm) interactions at lag grids 0–4, 01, 24, and 04.

### 2.8. House Heating Fuel

An additional analysis evaluated differences in the use of house heating fuel during the study period. The purpose of this analysis was to determine if there were differences in the type of house heating fuel used in urban grids with ambient air monitors versus rural grids without ambient air monitors. The 2005–2007 American Community Survey results were used [67,68]. House heating fuel estimates were available for jurisdictions with a population of at least 20,000 persons. Data were available for all six jurisdictions in urban grids with air monitors (Anne Arundel, Baltimore, Harford, Montgomery, and Prince George’s counties, and Baltimore city, MD, USA) and five of the jurisdictions in rural grids without air monitors. In rural grids without air monitors American Community Survey data were available for Frederick, Carroll, Howard, Cecil, and Talbot, but not for Kent counties.

### 2.9. Spatial Autocorrelations

The five fused surfaces (baseline PMB, PMC, PMCK, PMCQ, and PMCKQ) and ambient temperature, measured as *°*F, were evaluated using Moran’s *I* coefficient. This spatial autocorrelation analysis was accomplished using Proc Variogram in SAS/STAT software [78]. This statistic assesses spatial continuity between two spatial dimensions (CMAQ rows and columns in the Baltimore study area) by evaluating similarities between a selected outcome measure in multiple adjacent and distant observation pairs. Computed Moran *I* values are evaluated using the *Z* statistic. The *Z* statistic can be used to determine if the spatial autocorrelation is positive, negative, or absent, and yields a probability value. When the probability value indicates that there is a significant outcome, a positive *Z* value represents the presence of a positive autocorrelation, while a negative *Z* value represents a negative autocorrelation. The strength of the autocorrelation, positive or negative, is based on the size of the computed probability value, with a lower value representing a stronger autocorrelation.

## 3. Results

### 3.1. Correlations between Baseline PMB and Experimental AOD-PM_2.5_ Fused Surfaces

Table 4 displays correlations between baseline PMB and the four AOD-PM_2.5_ fused surfaces in all grids, and in grids with and without air monitors. Because there were many observations in the exposure dataset (Both = 8316; per grid monitor condition, Yes or No = 4158), all correlations were highly significant, all *p* ≤ 0.01. Nonetheless, there are interesting outcomes that provide clues as to the differential effectiveness of each of the AOD-PM_2.5_ fused surfaces over the baseline PMB fused surface in accurately estimating ambient fine PM concentration levels in the entire Baltimore study area, and in the two grid monitor conditions. First, correlations were highest between PMB and PMCQ in all grids, and in grids with and without air monitors. The percentage of shared variance, evaluated as a percentage value of the square of the correlation coefficient (*r*^*2%*^), was 94.7% for the Baltimore study area, 97.4% in grids with air monitors, and 94.3% in grids without air monitors. The *r*^2%^ (∆%) value of 3.1%, computed as the difference between grids with and without air monitors, was smallest for the PMCQ fused surface. PMC and PMCK AOD-PM_2.5_ fused surfaces had ∆% values of 32.4% and 35.7%, respectively. The PMCKQ ∆% value of 15.9% was between PMCQ’s lowest value and PMCK’s highest value.

#### 3.1.1. Fused Surface Means by Air Monitor Grid Condition

The three-year concentration level means for baseline PMB and the four AOD–PM_2.5_ fused surfaces in the three CMAQ air monitor grid conditions are presented in Table 5.

The first interesting observation concerns the fused surface with the highest mean concentration level in grids with and without air monitors: PMB in grids with air monitors (14.60 µg/m^3^), and PMCK in grids without air monitors (14.39 µg/m^3^). Second, the PMCK no monitor grid mean was not significantly different from PMCK monitor grid mean. These results suggest that the PMCK fused surface may provide a more stable concentration level estimate in grids with and without ambient air monitors.

#### 3.1.2. Fused Surface and Demographic Variable Categorical Analyses

Differences between experimental AOD-PM_2.5_ and baseline PMB fused surfaces and demographic variables for the three grid conditions are listed in Table 6.

The three-year mean (95% CI) for each fused surface for all 99 grids in the Baltimore study area were used to create three 95% CI categories. The **Below** category included all grid means (second column) that were below each fused surface 95% lower level value. The **Within** category included all grid means (second column) with values that were higher than the CI 95% lower level value and lower than the CI 95% upper level value. The **Above** category included all means (second column) that were higher than the CI 95% upper level value. As can be seen in Table 6, second column, all Chi-Square analyses for the three groups were significant (all *p’s ≤* 0.01). These results confirm that the three levels in each category group did not overlap and were thus unique. The remaining two columns in Table 6 show the results for each fused surface category group in grids with air monitors (third column) and in grids without air monitors (fourth column). In grids with air monitors, significant Chi-Square results only occurred for PMB and PMCQ fused surfaces (both *p’s* ≤ 0.01). For PMB and PMCQ fused surfaces, the category group totals and percentages were higher for the Above category than the Within or Below categories. In grids without air monitors, Chi-Square analyses were significant for all five fused surfaces (all *p’s* ≤ 0.01). Interestingly, for the five fused surfaces, the Above categories in grids without air monitors were significantly different from the Above categories in grids with air monitors (all *p’s* ≤ 0.01). For all five fused surfaces, the Above category totals in grids without air monitors were always larger than the Above category totals in grids with air monitors. Only the PMCK fused surface had both higher totals and percentages for the Above category in grids without air monitors than in grids with air monitors.

As can be seen at the bottom of Table 6, the same data analysis strategy as described above for the fused surfaces was implemented to analyze the two demographic variables, poverty percent and population density. There were minor but important differences in the results: Only the poverty percent category analysis in grids without air monitors was significant (*p* ≤ 0.05). For population density, the category analyses were significant in grids with air monitors (third column) and also in grids without air monitors (fourth column; both *p’s* ≤ 0.05). The poverty percent and population density Above categories analyses in grids without air monitors were not significantly different from grids with air monitors (both *p’s* > 0.05).

#### 3.1.3. Patient Characteristics

Table 7 shows totals and percentages for the four respiratory-cardiovascular ED visit and IP hospitalization groups stratified on case and control status and patients’ race. As seen in the Both column, second from left, ED asthma had the most observations (cases and controls combined, 47,256), and IP asthma had the fewest (13,515), with intermediate totals for MI (19,201) and HF (27,518). Totals and percentages for each of the four health outcome groups were higher in the 57 grids with no monitors than in the 15 grids with monitors. For each of the four health outcome groups, approximately 25% of observations were cases and about 75% were controls. The ratio of one case to three controls was also evident in grids with and without air monitors for ED asthma, IP asthma, MI, and HF (all *p’s* > 0.05). The ED asthma total (%) for Black patients (22,696, 48%) was higher than the totals (%) for White patients (21,294, 45%) and Other patients (3060, 6%). For the three IP groups, the totals (%) were highest for White patients, intermediate for Black patients, and lowest for Other patients. The race by CMAQ grid condition analyses were significant for all four health outcomes (all *p’s* ≤ 0.01). While there were more Black patients with ED asthma and IP asthma in grids with air monitors than in grids without air monitors, this relationship was reversed for Other patients and White patients. Black patients, Other patients, and White patients with IP MI or HF had higher totals (%) in grids without air monitors than in grids with air monitors.

### 3.2. CLR Analyses

CLR results showed significant differences between AOD-PM_2.5_ and baseline PMB fused surfaces, health outcome groups, lag grids, monitor conditions, and warm and cold season differences. For all CLR runs, ORs were significant at lag grids 0, 1, 01, and 04 (all *p’s ≤* 0.05). Effect modifiers were significant only for ED asthma in all grids (PMCQ: population density, lag grid 0, protective OR, *p ≤* 0.05), and in grids with monitors (PMB, PMC, PMCK, PMCQ, PMCKQ: season, lag grids 2–4, 04, all *p’s ≤* 0.05). By using the mean (95% CI), it was possible to evaluate OR magnitude differences for the four health outcomes, four AOD-PM_2.5_ and baseline PMB fused surfaces at lag grids 0, 1, 01, and 04.

#### 3.2.1. Emergency Department (ED) Asthma

**Figure 2.**
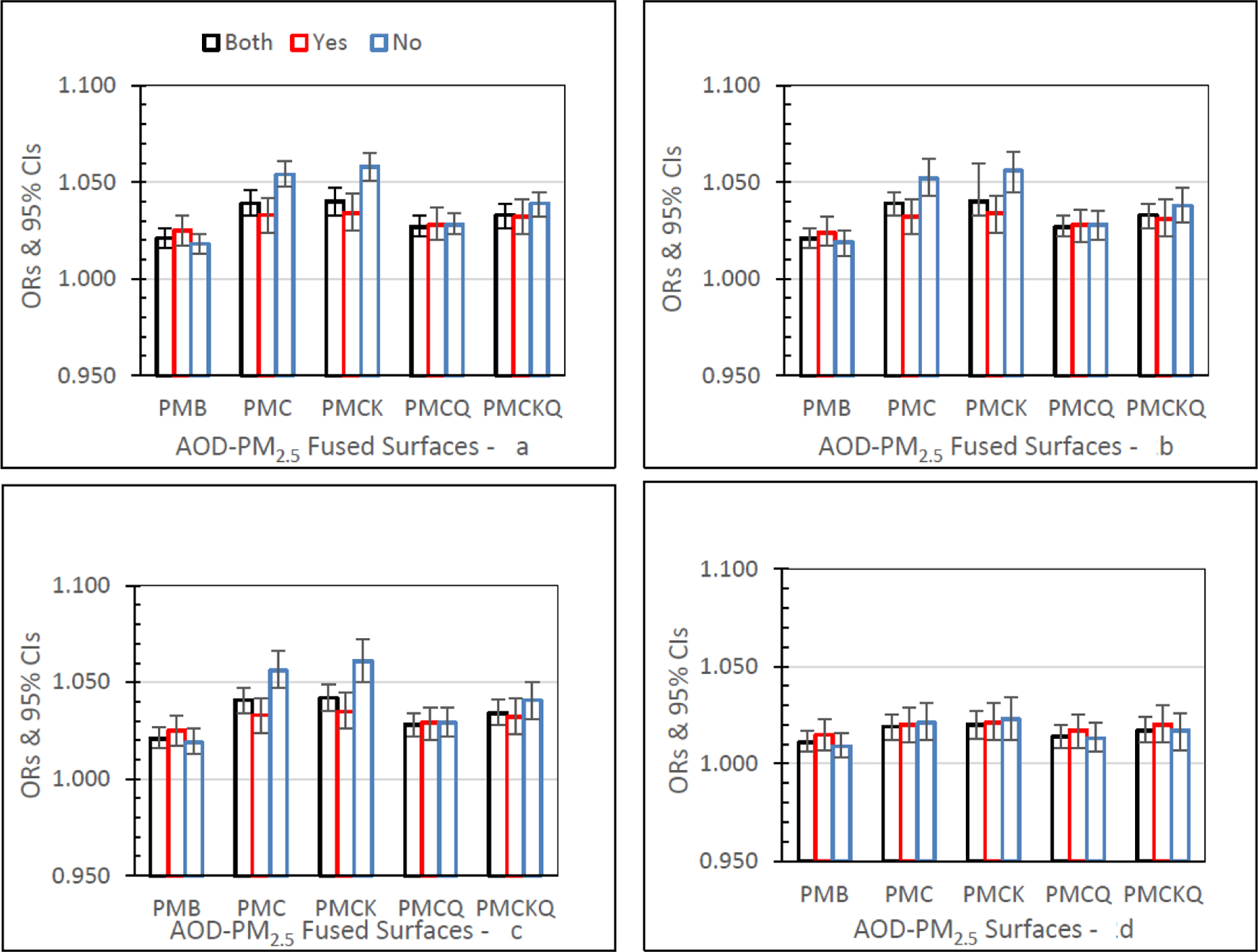
ED asthma ORs (95% CIs) for the four AOD-PM_2.5_ and baseline PMB fused surfaces, in all grids (Both), and in grids with (Yes) and without (No) air monitors, and in separate panels for lag grids 0 (**a**), 1 (**b**), 01 (**c**), and 04 (**d**).

Figure 2 shows ED asthma ORs (95% CIs) for the four AOD-PM_2.5_ and PMB fused surfaces. There are separate panels in Figure 2, one for each of the four lag grid values of 0, 1, 01, and 04. In the Both monitor grid condition, for lag grids 0, 1, and 01, ORs for each of the four AOD-PM_2.5_ fused surfaces were significantly higher than the baseline PMB ORs (all *p’s ≤* 0.05). In the Both monitor grid condition, for lag grid 04, the PMC, PMCK, and PMCKQ ORs were significantly higher than the PMB ORs (all *p’s ≤* 0.05). In lag grids 0, 1, and 01, the no monitor PMC and PMCK ORs were significantly higher than monitor PMC and PMCK ORs (all *p’s ≤* 0.05).

#### 3.2.2. Inpatient (IP) Asthma

**Figure 3.**
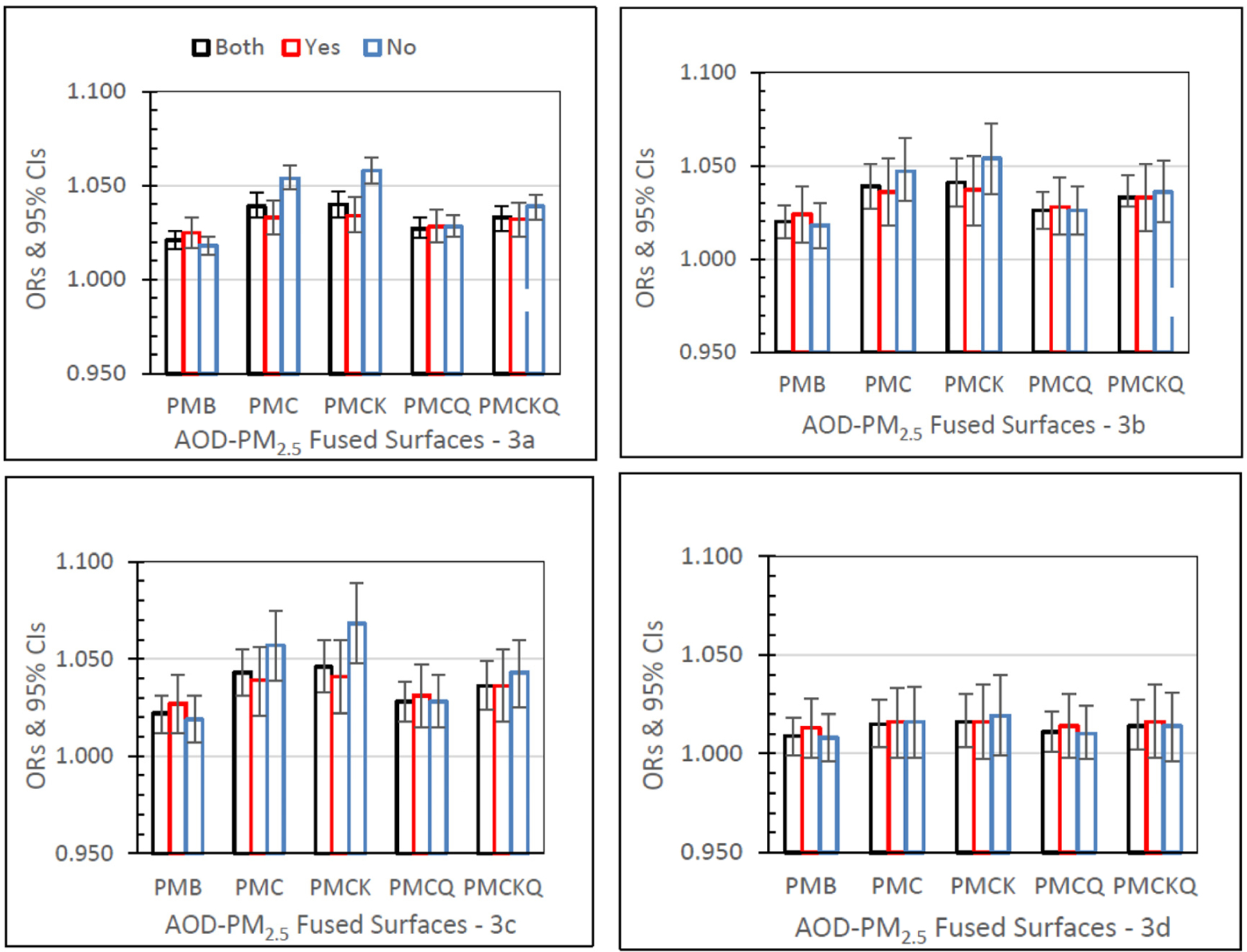
IP asthma ORs (95% CIs) for the four AOD-PM_2.5_ and baseline PMB fused surfaces, in all grids (Both), and in grids with (Yes) and without (No) air monitors, and in separate panels for lag grids 0 (**a**), 1 (**b**), 01 (**c**), and 04 (**d**).

Figure 3 shows IP asthma ORs (95% CIs) for the four AOD–PM_2.5_ and baseline PMB fused surfaces, and in separate panels for lag grids 0, 1, 01, and 04. In the Both monitor grid condition, for lag grids 0, 1, and 01, the PMC, PMCK, and PMCKQ ORs were significantly higher than the PMB ORs (all *p’s ≤* 0.05). Considering lag grids 0, and 01, only the no monitor PMCK ORs were significantly higher than the monitor PMCK ORs (both *p’s ≤* 0.05).

#### 3.2.3. Inpatient Myocardial Infarction (IP MI)

**Figure 4.**
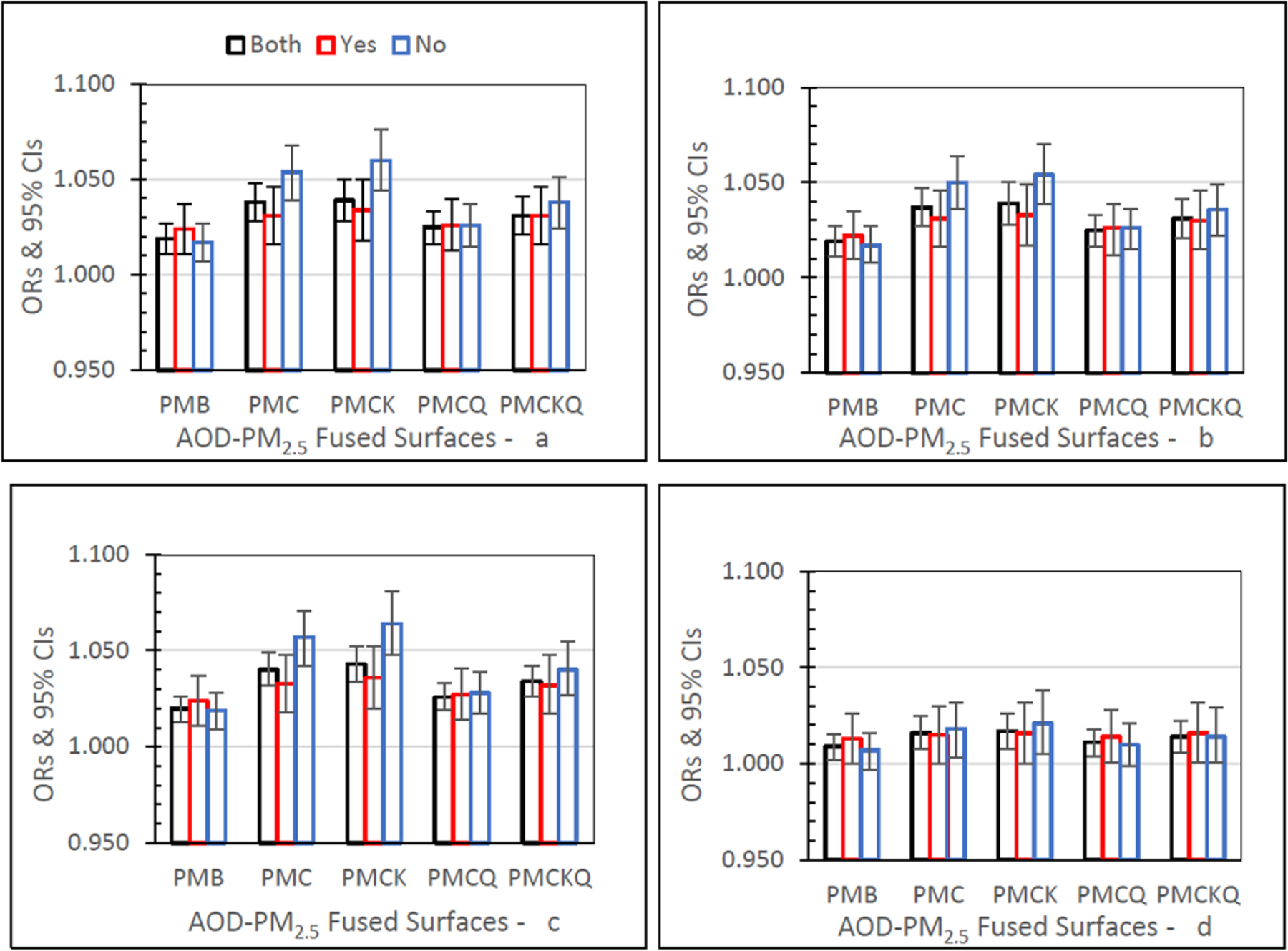
IP MI ORs (95% CIs) for the four AOD-PM_2.5_ and baseline PMB fused surfaces, in all grids (Both), and in grids with (Yes) and without (No) air monitors, and in separate panels for lag grids 0 (**a**), 1 (**b**), 01 (**c**), and 04 (**d**).

IP MI ORs for the four AOD–PM_2.5_ and baseline PMB fused surfaces, for lag grids 0, 1, 01, and 04, are displayed in Figure 4. In the Both monitor grid condition, for lag grids 0, 1, and 01, the PMC, PMCK, and PMCKQ ORs were significantly higher than PMB ORs (all *p’s ≤* 0.05). For lag grids 0, 1, and 01, the no monitor PMC and PMCK ORs were significantly higher than the monitor PMC and PMCK ORs (all *p’s ≤* 0.05).

#### 3.2.4. Inpatient Heart Failure (IP HF)

**Figure 5.**
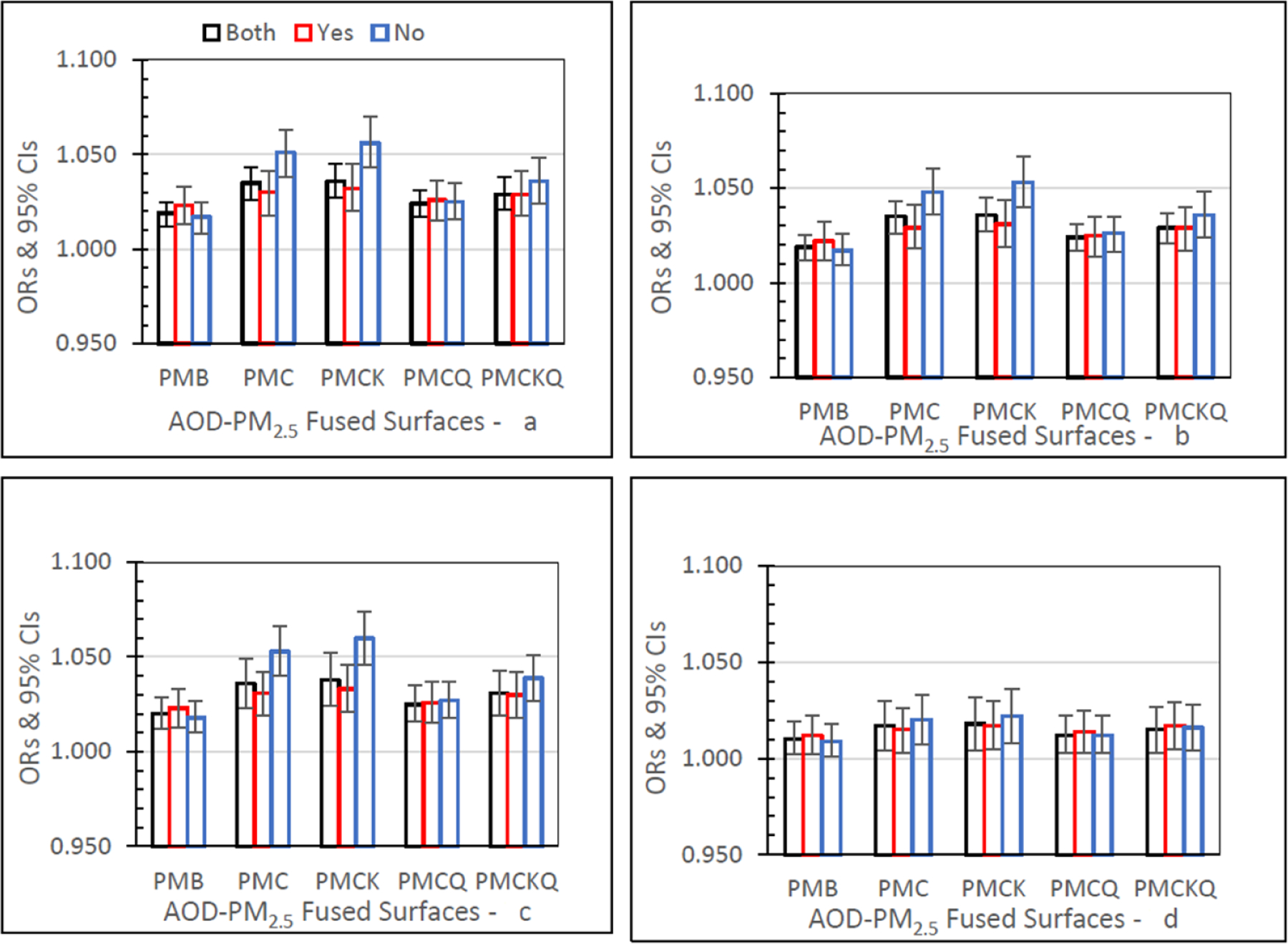
IP HF ORs (95% CIs) for the four AOD-PM_2.5_ and baseline PMB fused surfaces, in all grids (Both), and in grids with (Yes) and without (No) air monitors, and in separate panels for lag grids 0 (**a**), 1 (**b**), 01 (**c**), and 04 (**d**).

IP HF ORs for the five fused surfaces, three monitor grid conditions, and four lag grid values are displayed in Figure 5. In the Both monitor grid condition, for lag grids 0, 1, and 01, the PMC, PMCK, and PMCKQ ORs were significantly higher than the baseline PMB ORs (all *p’s ≤* 0.05). For the Both monitor grid condition, for lag grid 04, only the PMCK OR was significantly higher than the PMB OR (*p ≤* 0.05). In lag grids 0, 1, and 01, the no monitor PMC and PMCK ORs were significantly higher than monitor PMC and PMCK ORs (all *p’s ≤* 0.05).

### 3.3. No Monitor–Monitor OR Percent

**Figure 6.**
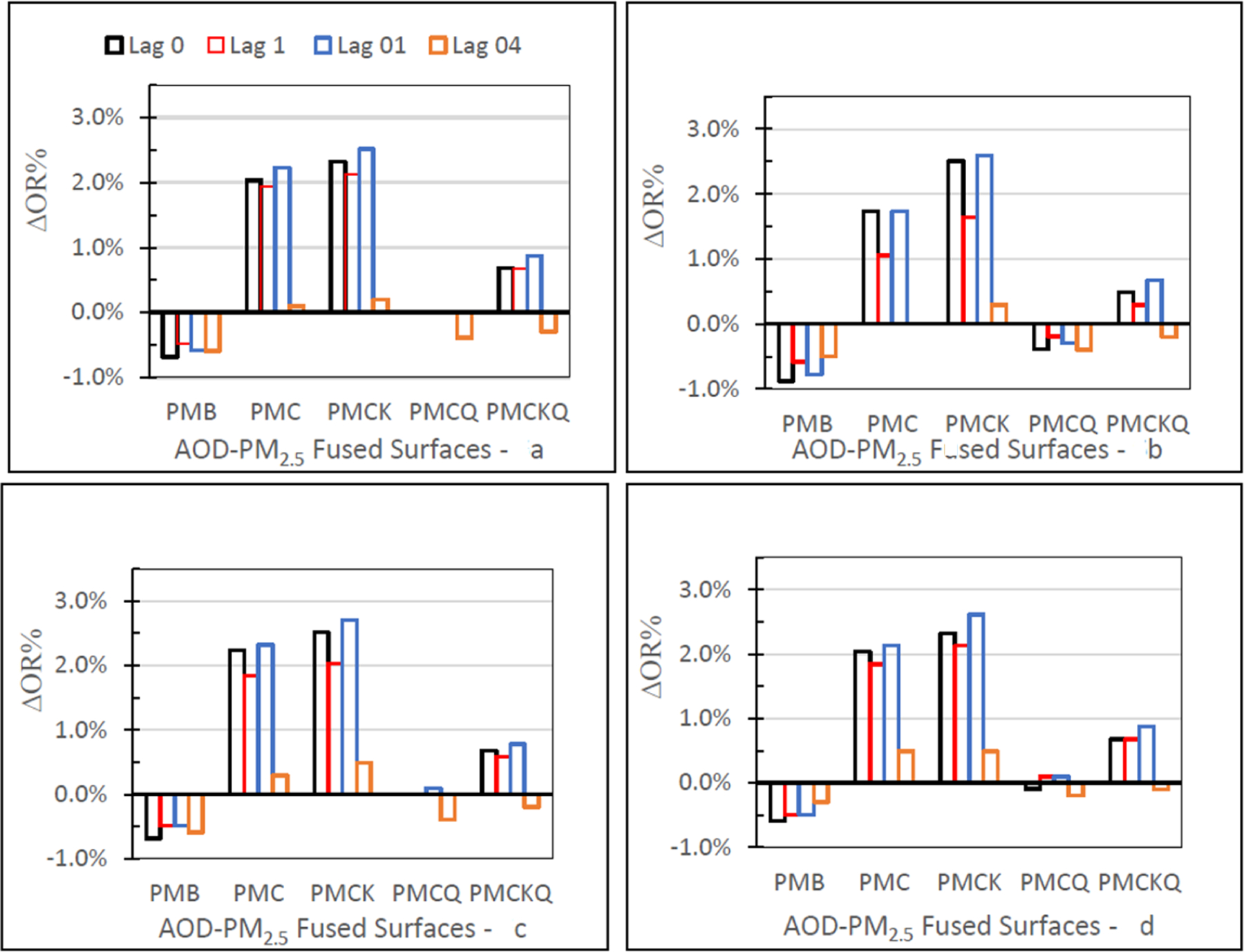
The no monitor–monitor OR percent (∆OR%) values for the four AOD-PM_2.5_ and baseline PMB fused surfaces for lag grids 0, 1, 01, and 04, and in separate panels for ED asthma (**a**), IP asthma (**b**), IP MI (**c**), and IP HF (**d**).

The no monitor-monitor OR percent (∆OR%) values for the four AOD-PM_2.5_ and baseline PMB fused surfaces and four lag grids 0, 1, 01, and 04, are displayed in Figure 6 in four separate panels, one for each of the four health outcomes. For the four health outcomes, ∆OR% values were larger for PMC and PMCK than the other three fused surfaces. These differences occurred for each of the four lag grid values. For the four health outcomes, PMC, PMCK, and PMCKQ fused surface ∆OR% values at lag 04 were smaller than the ∆OR% values at lag grids 0, 1, and 01. For the four health outcomes, PMB ∆OR% values were negative at lag grids 0, 1, 01, and 04. Results were mixed for PMCQ and PMCKQ. IP asthma PMCQ had negative ∆OR% values, as did IP asthma PMB. PMCKQ ∆OR% at lag grids 0, 1, and 01 were positive, resembling PMC and PMCK. One difference, however, was that the PMCKQ ∆OR% values were lower than the PMC and PMCK ∆OR% values at lag grid 04. PMCQ ∆OR% values were negative for ED asthma and IP asthma, resembling baseline PMB ED asthma and IP asthma ∆OR% values.

### 3.4. Size of Homogeneous Spatial Area

The largest size of the homogeneous spatial area (HOSA) was identified by the presence of a significantly higher AOD-PM_2.5_ fused surface OR than the baseline PMB OR for each chronic disease group (ED asthma, IP Asthma, MI, and HF) at lag grids 0, 1, 01, and 04. Table 8 summarizes the HOSA findings. For both monitor grid conditions combined (Both, top of Table 8), analyses of ED asthma and IP HF with the PMCK fused surface produced ORs that were significantly higher than the PMB ORs at lag grid values of 0, 1, 01, and 04 (all *p’s* ≤ 0.05). ORs for ED asthma with the PMC and PMCKQ fused surfaces were significantly higher than the PMB ORs at lag grids 0, 1, 01, and 04 (all *p’s* ≤ 0.05). Based on these significant outcomes at lag grid 04 for these three AOD-PM_2.5_ fused surfaces, it was concluded that the largest HOSA for PMC, PMCK, and PMCKQ fused surfaces was five grids (one 12 km-grid-wide × 5 grids) or 60 km. In grids with air monitors, only the ED asthma PMCK ORs were significantly higher than the PMB ORs at lag grids 0, 1, and 01 (all’s *p* ≤ 0.05). The width of the largest HOSA for PMCK was two grids wide, or 24 km. In grids without air monitors the analyses of ED asthma, IP MI, and HF with the PMC and PMCK fused surfaces produced ORs that were significantly higher than PMB ORs at lag grids 0, 1, 01, and 04 (all *p’s* ≤ 0.05). The analyses of ED asthma with PMCKQ resulted in ORs that were significantly higher than the PMB ORs at lag grids 0, 1, 01, and 04 (all *p’s* ≤ 0.05). The maximum size of the HOSA for PMC, PMCK, and PMCKQ was five grids wide, or 60 km.

#### Monitor–No Monitor Differences

The purpose of these analyses was to determine if the AOD-PM_2.5_ and baseline PMB fused surfaces had ORs that differed between grids without and with air monitors. Results are presented at the bottom of Table 8. The ED asthma, IP asthma, MI, and HF health outcomes evaluated with PMC and PMCK fused surfaces had ORs that were significantly higher in rural grids without air monitors than in urban grids with air monitors (all *p’s ≤* 0.05).

### 3.5. Warm–Cold Season Differences

There were warm and cold season differences for the four the AOD-PM_2.5_ fused surfaces and the four health outcomes (online resource includes electronic Supplementary Material consisting of Figure S1 (ED asthma: a-d, lag grids 0, 1, 01, 04, respectively) though Figure S4 (IP HF: a-d, lag grids 0, 1, 01, 04, respectively) for ED asthma, IP asthma, MI, and HF, respectively). These outcomes were expressed as larger ORs during the warm season and smaller ORs during the cold season. Specifics for each health outcome are presented below.

ED asthma (Figure S1), IP asthma (Figure S2), and MI (Figure S3) PMB, PMC, PMCK, PMCQ, and PMCKQ warm season ORs were significantly higher than cold season ORs at lag grids 0, 1, and 01 (all’s *p* ≤ 0.05). ED asthma, IP asthma, and MI PMCK warm season ORs were significantly higher than cold season ORs at lag grid 04 (all *p’s* ≤ 0.05). IP HF PMB, PMC, PMCK, PMCQ, and PMCKQ warm season ORs were significantly higher than cold season ORs at lag grids 0, 1, 01, and 04 (all *p’s* ≤ 0.05).

**Figure 7.**
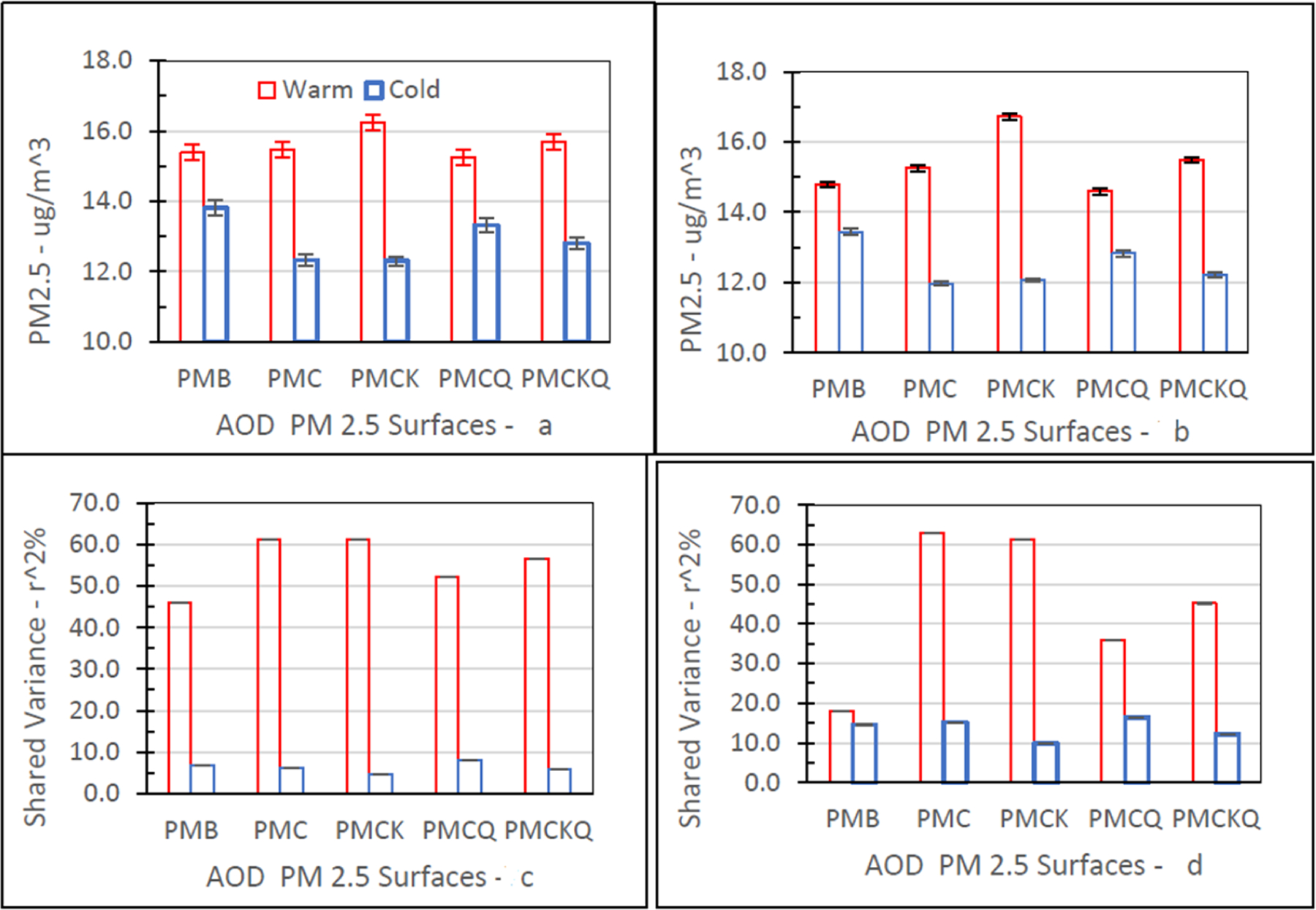
Warm–cold season differences for PM_2.5_ concentration (µg/m^3^; top: (**a**,**b**)) and shared variance percent between PM_2.5_ and temperature (*r*^*2%*^; bottom: (**c**,**d**)), in grids with (left: (**a**,**c**)) and without (right: (**b**,**d**)) ambient air monitors.

Figure 7 shows the three-year mean (95% CI) PM_2.5_ concentration levels for the four AOD–PM_2.5_ and baseline PMB fused surfaces and shared variance percent (*r*^2%^) between each fused surface and the three-year mean ambient temperature values in degrees *°*F by season (Warm, Cold) in grids with and without air monitors.

Fine PM concentration levels for the five PM_2.5_ fused surfaces were significantly higher during the warm season than the cold season, in grids with (top left panel, 6a) and without (top right panel, 6b) air monitors (all *p’s* ≤ 0.05). As expected, ambient temperatures during the warm season (Monitors, Yes: 70.61, 95% CI = 70.11–71.11; Monitor, No: 68.68, 95% CI = 68.46–68.89) were significantly higher than during the cold season (Monitor, Yes: 44.02, 95% CI = 43.54–44.51; Monitor, No: 42.53, 95% CI = 42.31–42.75) for the two monitor grid conditions (all *p’s* ≤ 0.05).

The measure of association shown is *r*^2%^, the square of the correlation (*r*) expressed as a percent. The *r*^2%^ value quantifies the percentage of the variance that is shared by each fused surface PM_2.5_ concentration level and ambient temperature value (*°*F). The *r*^2%^ values for the five fused surfaces were larger during the warm season than the cold season, especially in grids with air monitors (bottom left panel, 6c). The *r*^2%^ measures for PMC and PMCK were similar in grids with and without air monitors. For PMB, PMCQ, and PMCKQ, the magnitude of the warm season shared variance measure was lower in grids without air monitors than in grids with air monitors. For the five fused surfaces, the cold season *r*^*2%*^ values were higher in grids without air monitors than in grids with air monitors.

#### 3.5.1. Warm-Cold Season OR Percent

**Figure 8.**
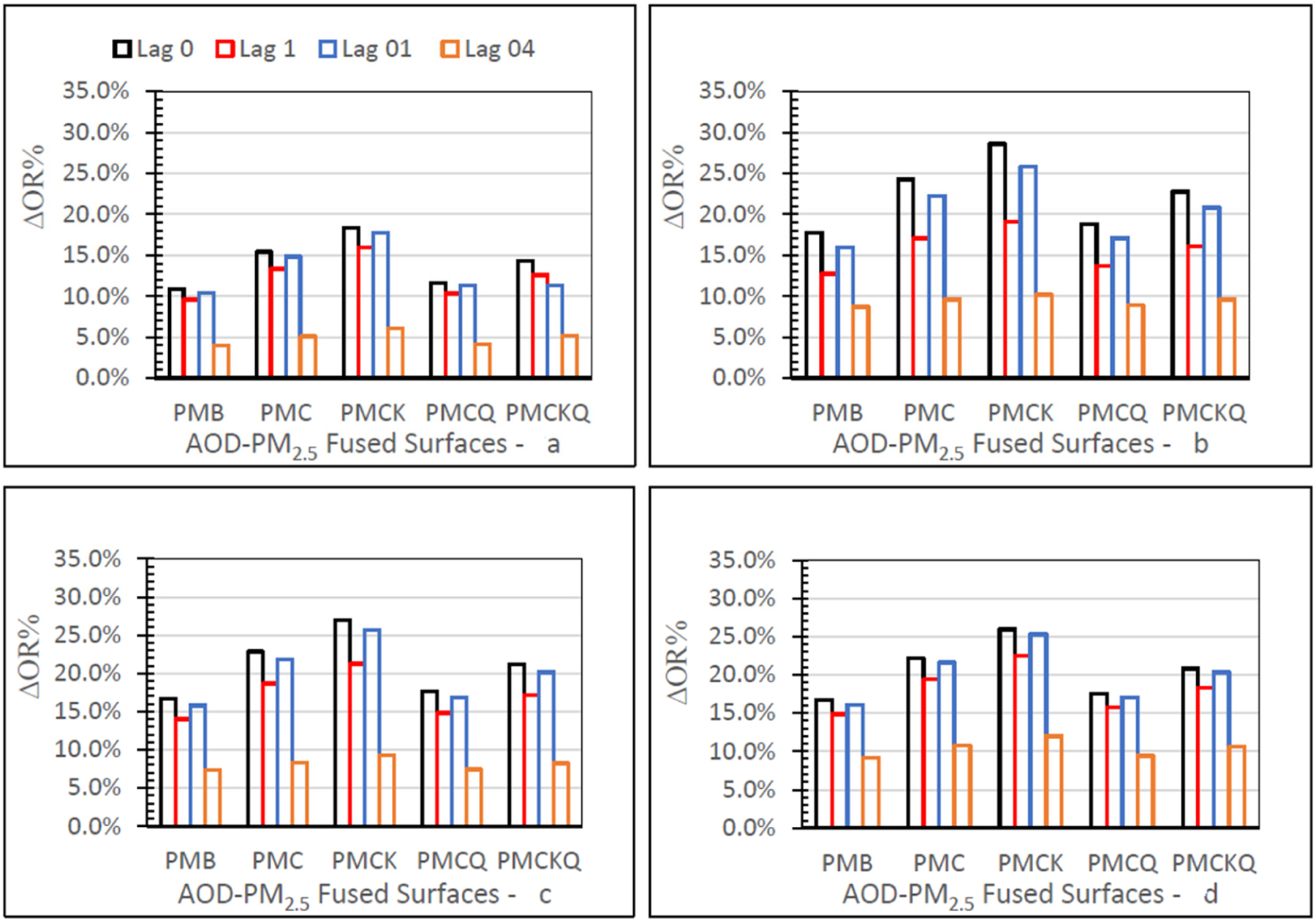
Warm-cold season ∆OR% values for the four AOD-PM_2.5_ and baseline PMB fused surfaces at lag grids 0, 1, 01, and 04 for ED asthma (**a**), IP asthma (**b**), IP MI (**c**), and IP HF (**d**), in different panels.

Figure 8 displays the warm–cold season odds ratio difference (∆OR%) values.

The ∆OR% measures were positive for each of the four AOD-PM_2.5_ and baseline PMB fused surfaces, ED asthma (panel a), IP asthma (b), MI (c), and HF (d), at lag grids 0, 1, 01, and 04. All four panels in Figure 8 demonstrate that PMC and PMCK had consistently higher ∆OR% values than the other two AOD-PM_2.5_ (PMCQ, PMCKQ) and baseline PMB fused surfaces at lag grids of 0, 1, and 01. ED asthma ∆OR% values were smaller than the ∆OR% values for IP asthma, MI, and HF. For all four health outcomes and the four AOD-PM_2.5_ and PMB fused surfaces, ∆OR% values were smaller at lag grid 04 than at the other three lag grid values of 0, 1, and 01.

#### 3.5.2. House Heating Fuel

As shown in Table 9, grids with (urban) and without (rural) ambient air monitors differed in the type of house heating fuel used between 2005 and 2007.

In urban grids (Yes), 56.4% of the houses surveyed used natural gas for heating—the most frequently used source of house heating fuel. In rural grids (No), 44.1% of the houses reported using electricity—the most used method to heat houses. Fuel oil (and kerosine) use was higher in grids without air monitors (17.9%) than in grids with air monitors (9.8%). The last row in Table 9 shows that between 2004 and 2006, the three-year mean ambient temperature (*°*F) was significantly higher in urban grids with air monitors (55.32) than in rural grids without air monitors (55.60) (*p ≤* 0.05).

#### 3.5.3. Spatial Autocorrelations in the Baltimore Study Area

Moran’s *I* statistic was used as a spatial summary measure to evaluate the presence of similar values in adjacent grids versus distant grids for the five fused surfaces and ambient temperature. These spatial autocorrelation analyses are in Table 10.

During the warm season, all *Z* values were positive and significant, thereby confirming the presence of positive autocorrelations between grid values and row-column grid distances (all *p’s* ≤ 0.01). During the cold season, only baseline PMB, PMCQ, and PMCKQ fused surfaces had significant and positive autocorrelation values (all’s *p* ≤ 0.01). Unexpectedly, during the cold season, the Moran *I* statistic was not significant for PMC and PMCK fused surfaces (both *p’s* > 0.05). When the same autocorrelation analyses were completed for all grids, however, all five fused surfaces, including PMC and PMCK, and ambient temperature again had significant and positive autocorrelation values (all *p’s ≤* 0.01).

### 3.6. Lag Grids versus Lag Days

There were similarities and differences between the spatial lag grid and temporal lag day monitor (Table S1, Supplementary Material) and season (Table S2, Supplementary Material) results. The no monitor-monitor odds ratio difference percent (∆OR%) values in Table S1 were the same for the five fused surfaces (PMB, PMC, PMCK, PMCQ, and PMCKQ) and the four health outcomes (ED asthma, IP asthma, MI, and HF) at lag grid 0, and lag day 0. The analysis of the four health outcomes with PMC and PMCK resulted in higher ∆OR% values at lag day 1 than at lag grid 1. Lag day 01 ∆OR% values were also higher than lag grid 01 ∆OR% values for PMC, PMCK, and PMCKQ with the four health outcomes. The combination of PMCQ with IP asthma and HF at lag value of 1, and PMCQ with IP asthma and MI at lag value of 01 resulted in lower ∆OR% lag day than lag grid ∆OR% values. Baseline PMB, in combination with the four health outcomes resulted in lower ∆OR% values at lag day 1 and 01 than at lag grid 1 and 01. Lower lag day than lag grid values also occurred for PMCKQ with ED asthma at lag day 1. There were significant lag grid values that included 0, 1, 01, and 04. Lag day values were significant for 0, 1, and 01, but not 04 (all *p’s ≤* 0.05). These outcomes occurred in grids with or without ambient air monitors in the spatial lag grid and temporal lag day analyses.

The ∆OR% measure was also used to evaluate warm-cold season differences obtained with the spatial lag grid analyses and the temporal lag day analyses. The ∆OR% lag grid and lag day values were the same for the five fused surfaces and four health outcomes at lag value of 0. For the five fused surfaces and four health outcomes at lags of 1 and 01, all ∆OR% values were higher for lag days than lag grids. Only the warm season ORs were significant for some of the lag grid and lag day analyses. PMCK identified more significant warm season ORs in the lag grid and lag day analyses than the other three AOD-PM_2.5_ fused surfaces (all *p’s ≤* 0.05). There were non-significant warm season ORs for baseline PMB in combination with the four health outcomes (all *p’s* > 0.05). The other difference was due the length of the lag grid values versus the lag day values, with the former having significant ORs for all four lag grids (0, 1, 01, and 04), while the latter only had significant ORs for three of the four lag days (0, 1, 01).

## 4. Discussion

This study implemented, for the first time, a spatial lag grid analysis of Hierarchical Bayesian Model (HBM) assembled experimental AOD-PM_2.5_ and baseline PMB fused surfaces and respiratory-cardiovascular ED visits and IP hospitalizations with patient residences uniquely assigned to 12 km^2^ CMAQ grids. Because the spatial lag grid analyses were completed using the same concatenated exposure-health outcome data files that were previously used to evaluate temporal lag days [3], it was possible to identify unique differences and similarities associated with these two case-crossover data analysis procedures. Unique findings include a description and interpretation of differences among the size of homogeneous spatial areas (HOSAs), greater risk of manifesting a respiratory-cardiovascular chronic disease as a result of exposure to higher AOD-PM_2.5_ concentration levels in rural areas, and warm-cold season differences that showed associations between ambient temperature and AOD-PM_2.5_ concentration levels, and increases in respiratory-cardiovascular chronic disease ED visits for asthma and IP hospitalizations for asthma, MI, and HF. Each of these four topics are detailed below after reviewing correlations and descriptive statistical analyses for baseline PMB and the four experimental AOD-PM_2.5_ fused surfaces.

Correlations and three-year fine PM concentration level means suggest that PMCK may be more representative of ambient PM_2.5_ concentration levels in urban grids with air monitors and in rural grids without air monitors than baseline PMB, or the other three AOD-PM_2.5_ fused surfaces. The percentage decrease in shared variance between PMB-PMC and PMB-PMCK in grids with and without air monitors was 32.4% and 35.7%, respectively. In grids without air monitors, the baseline PMB concentration levels reflect less emphasis on air monitor readings and greater importance on CMAQ PM_2.5_ model estimates. In grids without air monitors, PMC uses AOD-PM_2.5_ readings (with missing values) while PMCK utilizes Kriged AOD_K-_PM_2.5_ readings (without missing values) to estimate ambient fine PM concentrations. One interpretation could be that in grids without air monitors, AOD-PM_2.5_ concentration levels are more representative of ambient monitor PM_2.5_ measurements than the Community Multiscale Air Quality (CMAQ) PM_2.5_ model estimates.

The first aim was to determine the size of homogeneous spatial areas, HOSAs. HOSA sizes in km for width and height or km^2^ for area differed as a function of AOD-PM_2.5_ fused surface, respiratory-cardiovascular ED visits or IP hospitalizations, and monitor grid condition. The largest HOSAs included 5 interconnected grids (720 km^2^), and the smallest HOSAs were two grids wide (288 km^2^). In grids with monitors, there was only one HOSA two grids wide for PMCK and ED asthma. In grids without air monitors, there were seven HOSAs five grids wide: three each for PMC or PMCK paired with ED asthma, IP MI, or HF, and one for PMCKQ with ED asthma. The grid sizes of HOSAs in grids without monitors were identical to the grid sizes of HOSAs in the Baltimore study area for PMCKQ, and similar for PMC and PMCK. In the Both grid monitor condition, three HOSAs five grids wide occurred for PMC with ED asthma, and PMCK with ED asthma or IP HF. These maximum grid size HOSA results suggest that the benefit of utilizing the experimental AOD-PM_2.5_ fused surfaces may be only evident when the analyses are completed in rural grids without air monitors. The other implication is that the same association between an experimental AOD-PM_2.5_ fused surface and a specific respiratory-cardiovascular ED visit or IP hospitalization outcome can occur up to 60 km from lag grid 0. It is possible that residents in these five interconnected rural grids without air monitors were at equal risk of exposure to higher fine PM concentration levels and in need of medical care for ED asthma or an overnight stay in the hospital for MI or HF. Residents in two interconnected rural grids without air monitors were also at risk of exposure to higher fine PM concentration levels and in need of medical care as an IP because of the onset of uncontrolled asthma attacks.

The second objective concerned the presence of differences in AOD-PM_2.5_ concentration levels between urban grids with air monitors and rural grids without air monitors. The contribution of PMC or PMCK concentration levels to ED asthma, IP asthma, MI, and HF produced significantly higher ORs in grids without air monitors than in grids with air monitors at lag grids 0, 1, and 01. Interestingly, the no monitor–monitor odds ratio difference percent (∆OR%) values were positive and larger for PMC and PMCK than for PMCKQ fused surfaces. The ∆OR% values were larger at lag grids 0, 1, and 01 than at lag grid 04. It is possible that the PMC and PMCK concentration levels were more accurate approximations of ambient fine PM values in urban grids with air monitors and in rural grids without air monitors than the PMCKQ concentration levels. Because the number and percentage of true positive cases and true negative controls were similar in urban grids with air monitors and in rural grids without air monitors, we concluded that the concentration–response function between AOD-PM_2.5_ concentration levels and respiratory-cardiovascular ED visits and IP hospitalizations could be similar in the entire Baltimore study area, and in urban grids with air monitors and in rural grids without air monitors [80]. One limitation, however, is that this statement is based on a temporal lag day analysis and does not utilize the spatial lag grid case-crossover design analysis.

Categorical analyses of the Baltimore study area identified, for the first time, a subset of homogeneous spatial grids in rural areas without ambient air monitors that resembled urban grids with ambient air monitors by showing higher poverty percent and increased population density. In rural areas without air monitors, there were eight grids with poverty percent values in the Above category, and seven grids in urban areas with air monitors that had poverty percent values in the Above category. There were 19 rural grids with population density in the Above category and 11 urban grids in the Above category. In addition, there were higher totals and percentages of IP MI (12,016, 62.6%) and IP HF (15,684, 57.0%) in rural areas than in urban areas (7185, 37.4% and 11,834, 43.0%, respectively). The totals and the percentages of Black ED asthma patients (11,844, 25.2%) and IP asthma patients (2358, 17.5%) were higher in urban areas than in rural areas (10,852, 23.1% and 2152, 16.0%, respectively). These results support the conclusion that persons living in rural grids are also at risk of developing respiratory-cardiovascular chronic diseases after exposure to higher fine PM concentration levels, as are persons who reside in urban grids.

Hirshon and associates [81] evaluated PM_2.5_ zinc concentration levels and children’s asthma ED visits and IP hospitalizations. These authors reported that PM_2.5_ zinc concentrations contributed to increases in asthma hospital events. This publication did not identify the zinc source, however. One explanation could be that the ambient PM_2.5_ zinc levels recorded at the Baltimore PM_2.5_ Supersite [82] may have come, in part, from a nearby Toxic Release Inventory site [83]. The Baltimore PM_2.5_ Supersite location can be remapped onto one of the 99 CMAQ grids utilized in the Baltimore study area. As a result of this remapping, we discovered that the location of the Baltimore PM_2.5_ Supersite is in CMAQ grid R6, C6. This same grid also had three federal reference method PM_2.5_ air monitors and one U.S. Environmental Protection Agency-identified Toxic Release Inventory facility that emitted ambient zinc fumes and dust. An adjacent Baltimore study area grid, R_6_, C_7_, had one ambient PM_2.5_ air monitor and one Toxic Release Inventory facility that released zinc fumes and dust in the air.

As a follow-up to the Hirshon et al. study [81], we also looked at the number of Toxic Release Inventory facilities that released zinc fumes or dust in the Baltimore study area between 2004 and 2006. There were five different businesses operating during this three-year timeframe. One company had facilities in two distinct locations, contributing a total of 11 zinc point sources: seven zinc fumes or dust point sources in grids with air monitors (urban grids) and four in grids without air monitors (rural grids). This analysis suggests that the ambient PM_2.5_ zinc measured at the Baltimore PM_2.5_ Supersite in 2002 could have originated from a nearby Toxic Release Inventory facility that emitted zinc fumes and/or dust. Although the federal reference method PM_2.5_ air monitors data we utilized did not include PM_2.5_ zinc measurements, it is possible that ambient zinc from Toxic Release Inventory zinc-emitting facilities in selected at-risk grids (with or without air monitors) in the Baltimore study area could have indirectly contributed to children’s asthma ED visits and IP hospitalizations.

Although this study did not evaluate environmental hazards associated with living close to brownfields [84,85] or U.S. Environmental Protection Agency Toxic Release Inventory facilities [83], several published studies have described the environmental contamination from manufacturing efforts in Maryland that have included U.S. Environmental Protection Agency Toxic Release Inventory facilities in the state [86–89] and brownfields in south Baltimore [84,85]. Perlin, Sexton, and Wong [87] found that there were 122 Toxic Release Inventory sites in Maryland. About half of the Toxic Release Inventory facilities were in Baltimore city, Howard, Anne Arundel, and Baltimore counties. Maryland residents near a Toxic Release Inventory site were medically underserved [89]. South Baltimore brownfields have higher respiratory and heart disease mortality rates among White working-class residents than the rest of Baltimore city and state [84]. Litt, Tran, and Burke [85] described a variety of environmental hazards in southeast Baltimore that included heavy metals, solvents, and insecticides. Living in these environmentally compromised areas for long durations could increase residents’ adverse responsiveness to lower ambient PM_2.5_ concentration levels and their enhanced contribution to respiratory-cardiovascular ED visits or IP hospitalizations [84,85,90,91].

The third aim was to spatially evaluate warm–cold season differences. For the Baltimore study area, there were significant correlations between ambient temperature and fused surface concentration levels. The percentage of shared variance was highest between ambient temperature and PMCK in all three grid conditions. After controlling for experienced apparent temperature in the CLRs, only the PMCK AOD-PM_2.5_ fused surface had the highest number of significant ORs, thereby representing a greater risk of warm season effects on the occurrence of respiratory-cardiovascular ED visits and IP hospitalizations at lag grids 0 (all four health outcomes), 1 (ED asthma, IP MI, and HF), 01 (all four health outcomes), and 04 (IP asthma, MI, and HF). The PMCK warm-cold season odds ratio difference percent (∆OR%) values were smallest at lag grid 04 compared to lag grids 0, 1, and 01. Unlike these spatial lag grid warm-cold season results, the temporal lag day outcomes had (1) no significant lag day 04 and (2) higher ∆OR% values for the five fused surfaces and four health outcomes at lag days 1, and 01. These new findings suggest that the warm-cold season differences depend on what AOD-PM_2.5_ fused surface is used and the implementation of the spatial lag grid analysis or temporal lag day analysis.

Additional results dealing with season differences appear to increase our understanding of the complex relationship between ambient pollution sources and their synergistic interaction with house heating fuel type, urban-rural environments, ambient temperature, and AOD-PM_2.5_ fused surface characteristics. During the cold season, all correlations between ambient temperature and PMB AOD-PM_2.5_ concentration levels were significant and negative. This inverse correlation suggests that higher fine PM levels were associated with lower ambient temperatures. For the five fused surfaces, the percent of shared variance (*r*^*2%*^) measures were higher in rural grids without air monitors than in urban grids without air monitors. Three-year ambient temperature means were significantly higher in urban grids with air monitors than in rural grids without air monitors during the warm and cold seasons. The use of oil, kerosine, or similar carbon-based products as a home heating fuel was higher in rural grids without air monitors than in urban grids with air monitors. The use of oil as a house heating fuel could be another unique contributor to the documented elevated AOD-PM_2.5_ concentration levels in rural grids without air monitors. Finally, spatial autocorrelation analyses showed the absence of a positive relationship only for PMC and PMCK fused surfaces during the cold season.

The last aim was to compile this study’s findings regarding differences between the spatial lag grid case-crossover method used in this report and the temporal lag day case-crossover method previously implemented in an earlier publication [3]. There were more differences than similarities between lag grids and lag days. First, the longest lag day was two (lag day 01), while the longest lag grid was five (lag grid 04). Second, the longer lag grid (0, 1, 01, 04) and shorter lag day (0, 1, 01) values were also found for the monitor and season analyses. Third, the no monitor-monitor odds ratio difference percent (∆OR%) lag grid values were either the same, higher for lag days, or higher for lag grids based on what fused surface was used to analyze a specific respiratory-cardiovascular chronic disease. Fourth, the warm-cold season ∆OR% lag grid values were either the same (lag value 0) or higher for lag days than lag grids (lag values 1, or 01). Fifth, the lag grid and lag day analyses showed that PMCK identified more significant warm season ORs than either PMC or PMCKQ.

Work on the adverse effects of ultrafine PM on a variety of physiologic measures and health outcomes has continued for three decades, but it is now becoming more relevant given the unequivocal evidence of the detrimental effects of ambient and modeled fine PM on many health outcomes [28]. Mechanistically, ultrafine PM’s adverse effects should be even more severe than fine PM’s adverse effects on the occurrence of respiratory-cardiovascular chronic diseases [21,22,24,25,27–29,92]. It is possible that technical issues related to the use of the selected data analytic methods could explain why there have not been more epidemiologic studies reporting on the significant association between ultrafine PM and respiratory-cardiovascular outcomes [93–95]. Other possibilities could include the ambient ultrafine PM air monitor location and its distance from the residential addresses of study participants [19,26,93,94]; the type of study participant selected with pre-morbid conditions that would enhance the adverse effects of ultrafine PM on the occurrence of a respiratory-cardiovascular chronic disease [20,26,27,29]; and that ultrafine PM’s adverse physiologic effects could compromise other organs besides the lungs and heart, e.g., liver, kidney, brain, and this type of system-wide structural damage and physiologic modification could facilitate the development of respiratory-cardiovascular chronic diseases at a later date [22,23,26,28].

The suggestion introduced here is that the accuracy of AOD-PM_2.5_ concentration levels in estimating ambient monitor PM_2.5_ measurements is related to spatial scale [38,96–99]. Remote sensing studies that have used AOD to estimate ambient PM_2.5_ measurements have concluded that smaller grids provide greater accuracy than larger grids. We utilized the National Aeronautics and Space Administration 10 km^2^ grid when we accessed the AOD unitless readings for the Baltimore and New York City study areas. Since our initial objective was to evaluate the accuracy of the four experimental AOD–PM_2.5_ concentration level fused surfaces relative to the performance of the previously developed baseline PMB, we decided to map the concentration level values to CMAQ’s 12 km^2^ native grid system before the Baltimore and New York City epidemiologic studies were undertaken. For the Baltimore and New York City study areas, we controlled for scale effects by using the CMAQ 12 km^2^ grid system as the smallest spatial area of analysis. In the Baltimore study analyses, all data files with different spatial polygons, Zone Improvement Plan (ZIP) codes for ED visits and IP hospitalizations, and ZIP Code Tabulation Areas (ZCTAs) for population density and poverty measures were mapped to CMAQ grids. In subsequent analyses, the CMAQ grids were categorized into one group including air monitors (urban grids), and another group without air monitors (rural grids). The larger groups, consisting of urban grids with air monitors or rural grids without air monitors, should not be less accurate than the individual 12 km^2^ grids since the larger areas represent the inclusion of 12 km^2^ grids into larger spatial grid areas. Likewise, the accuracy of the identified homogeneous spatial areas (HOSAs) and heterogenous spatial areas should have the same accuracy as each of the individual Community Multiscale Air Quality (CMAQ) grids.

There are strengths in the analytical methods utilized in this spatial lag grid case-crossover study. To our knowledge, this is the first time the temporal lag day case-crossover design has been modified to evaluate lag grids. The selection of controls that preceded or followed the cases makes the lag grid case-crossover design spatially bidirectional [66]. The spatial lag grid analytic method permits the identification and assessment of homogeneous spatial areas (HOSAs) and heterogeneous spatial areas. The study results demonstrate, also for the first time, the presence of rural grids without air monitors that resembled urban grids with air monitors in exposing rural residents to increased fine PM concentration levels and seeking medical care for respiratory-cardiovascular chronic disease hospital events. A final contribution was confirmation that PMCK could become a replacement for baseline PMB and its use in epidemiologic studies to evaluate the contribution of AOD–PM_2.5_ concentration levels on the future occurrence of respiratory-cardiovascular ED visits and IP hospitalizations in urban grids with ambient air monitors and in rural grids without ambient air monitors.

There are unresolved methodological issues that limit the generalizability of these study results. The 99 CMAQ grids that defined the Baltimore study area included 15 urban grids with air monitors and 84 rural grids without air monitors. All 15 urban grids with monitors had associated respiratory–cardiovascular hospital event data. Only 57 of the 84 rural grids without air monitors had respiratory-cardiovascular hospital event data. Grids without air monitors that lacked health data could have included some of those grids over the Chesapeake Bay. The Chesapeake Bay CMAQ grids, located in the south-east corner of the Baltimore study area, included more water than land mass. Residents live on part of Maryland’s irregular coastline and islands. Underestimates for total patients with respiratory-cardiovascular ED visits and IP hospitalizations could have occurred because some Maryland residents can and do obtain medical treatment out of state, e.g., in Washington, DC; Virginia; or Pennsylvania. To be consistent with the way the linear boundaries for the New York City study area and the Baltimore study area were established for the purpose of developing the AOD–PM_2.5_ and PMB fused surfaces, it was necessary to include all 99 CMAQ grids in the Baltimore study area. There were boundary grids that crossed the Maryland state line into neighboring states. It was not possible to identify which grids were included in homogeneous spatial areas (HOSAs). Based on the way the spatial lag grid analytical method was implemented, only HOSA size could be determined. There was no independent confirmation of actual ambient PM_2.5_ concentration levels in rural grids without air monitors. There was also (relative) spatial heterogeneity in the 15 urban grids with air monitors because there were only 17 ambient air monitors for an area of (12 km x 12 km x 15 grids) 2160 km^2^. If the 17 fine PM ambient air monitors were equally distributed among the 15 CMAQ grids, each monitor grid would occupy 127 km^2^. Monitor accuracy for the fine PM measurements is highest at the monitor’s location. Fine PM measurement accuracy decreases as the distance from the monitor increases [8,33]. It is possible that ambient PM_2.5_ concentration levels in the most distant grid with a lag grid value of 4 was the same at the ambient PM_2.5_ concentration level in lag grid 0. It is not clear to what extent health care access impacted the results. Available evidence indicates that this may not have been a bias since, for some respiratory-cardiovascular chronic diseases included in the Baltimore study area, there were more patients with the four respiratory-cardiovascular chronic diseases in rural grids without air monitors than in urban grids with air monitors.

Future research efforts should involve the identification of criteria that will lead to the replacement of the currently used baseline PMB with another AOD-PM_2.5_ fused surface, e.g., PMCK. Relevant attributes that could facilitate the selection of an updated AOD-PM_2.5_ baseline could include grid resolution below 10 km^2^, absence of missing AOD unitless readings, and improved accuracy of PM_2.5_ concentration levels in estimating ambient fine PM concentration levels in grids without ambient air monitors. In addition, to increase the reliability and validity of AOD-PM_2.5_ fused surface concentration levels in grids without air monitors, it will be necessary to have available, independent, on-the-ground ambient PM_2.5_ measurements in grids without ambient air monitors. This more ambitious goal could be reached by using portable and accurate PM_2.5_ monitors to supplement fine PM readings available from the U.S. Environmental Protection Agency Air Quality System national network of ambient air monitors [50]. In addition, ultrafine PM monitoring should be included along with fine PM monitoring within selected communities in urban and rural areas. The overall goal of these proposed improvements is to protect the respiratory-cardiovascular health of residents from the adverse consequences of breathing ambient air with elevated fine PM and ultrafine PM concentration levels wherever they live, in urban or rural areas of Maryland or other states in the U.S.

## 5. Conclusions

The spatial lag grid case-crossover results provide support for the use of this new analytical method to identify homogeneous spatial areas (HOSAs), areas that demonstrated a similar relationship between elevated AOD-PM_2.5_ fused surface concentration levels and increased respiratory-cardiovascular hospital ED visits and IP hospitalizations in the entire Baltimore study area, in urban grids with air monitors, and in rural grids without air monitors. With the PMC and PMCK fused surfaces, the largest homogeneous spatial area (HOSA) was 720 km^2^ for the Baltimore study area and in rural grids without air monitors. Some rural grids without air monitors resembled urban grids with air monitors in the contribution of increased AOD-PM_2.5_ fused surface concentration levels to the occurrence of health outcomes evaluated. Results from categorical data analyses identified a subset of rural grids without air monitors that also demonstrated higher poverty percent, increased population density, and elevated AOD-PM_2.5_ concentration levels, as was found for urban grids with air monitors. Warm-cold season analyses showed that elevated AOD-PM_2.5_ concentration levels during the warm season contributed to increases in respiratory-cardiovascular ED visits and IP hospitalizations, especially when the PMCK fused surface was used. New information confirmed the association between elevated AOD-PM_2.5_ concentration levels and ambient temperature. PMC and PMCK fused surfaces consistently demonstrated greater differences between warm and cold seasons than the other two AOD-PM_2.5_ fused surfaces that included Community Multiscale Air Quality (CMAQ) PM_2.5_ estimates (PMCQ and PMCKQ), or the currently used baseline, PMB. Obtained differences or similarities between spatial lag grid and temporal lag day analyses varied based on the type of AOD-PM_2.5_ fused surface selected, the specific respiratory-cardiovascular chronic disease utilized, and what lag value was evaluated. Future research efforts should continue to evaluate the contribution of increased ambient fine PM (as well as ultrafine PM) levels and area-specific demographic and environmental hazards and their contribution to increased susceptibility among persons developing respiratory–cardiovascular chronic diseases and residing in Maryland’s rural areas, or in rural areas in other locations.

## Supplementary Material

Supplement1 Figure S1: ED asthma ORs and 95% CIs for the four AOD-PM_2.5_ and baseline PMB fused surfaces during the warm and cold seasons at lag grids 0 (a), 1 (b), 01 (c), and 04 (d). Figure S2: IP asthma ORs and 95% CIs for the four AOD-PM_2.5_ and baseline PMB fused surfaces during the warm and cold seasons at lag grids 0 (a), 1 (b), 01 (c), and 04 (d). Figure S3: IP MI ORs and 95% CIs for the four AOD-PM_2.5_ and baseline PMB fused surfaces during the warm and cold seasons at lag grids 0 (a), 1 (b), 01 (c), and 04 (d). Figure S4: IP HF ORs and 95% CIs for the four AOD-PM_2.5_ and baseline PMB fused surfaces during the warm and cold seasons at lag grids 0 (a), 1 (b), 01 (c), and 04 (d). Table S1: No monitor–monitor ∆OR% for the four AOD-PM_2.5_ and baseline PMB fused surfaces and the four respiratory-cardiovascular ED visits and IP hospitalizations lag grid and lag day analyses. Table S2: Warm-cold season ∆OR% for the four AOD-PM_2.5_ and baseline PMB fused surfaces and four respiratory-cardiovascular ED visits and inpatient hospitalizations lag grid and lag day analyses.

## Figures and Tables

**Table 1.** Bidirectional spatial lag grid case-crossover analyses for the 99 12 km^2^ CMAQ grids with (red) and without (dash, ‘-’) ambient PM_2.5_ air monitors in the Baltimore study area ^1*−*3^.

Community Multiscale Air Quality (CMAQ) Grid Columns								
1	2	3	4	5	6	7	8	9
**(11,1)**	-	-	-	**N (11, 5)**	-	-	-	**(11, 9)**
-	-	-	-	-	-	-	-	-
-	-	-	-	-	-	-	-	-
-	-	-	-	-	(8, 6/B)	-	(8, (8, 8/H))	-
-	-	-	-	(7, 5/BC)	(7, 6/BC)	(7, 7/B)	-	-
**W (6,1)**	-	-	-	-	(6, 6/BC)	(6, 7/BC)	-	**E (6, 9)**
-	-	-	-	-	(5, 6/AA)	(5, 7/AA)	-	-
-	-	(4, 3/M)	-	(4, 5/PG)	(4, 6/AA)	-	-	-
-	-	-	-	(3, 5/PG)	-	(3, 7/AA)	-	-
-	-	-	-	-	(2, 6/PG)	-	-	-
**(1, 1)**	-	-	-	**S (1, 5)**	-	-	-	**(1, 9)**

1 CMAQ grid coordinates, shown in black and bold font, are 1–11 (S to N) rows and 1–9 (W to E) columns.

2 Grid coordinates, displayed in red hue and in bold font, had one or more ambient federal reference method PM_2.5_ air monitors. One urban grid (**R**_**6**_, **C**_**6**_) in Baltimore city had three different PM_2.5_ air monitors. The other 14 urban grids with air monitors had one ambient PM_2.5_ air monitor per grid.

3 Abbreviations: AA = Anne Arundel county; B = Baltimore county; BC = Baltimore city; H = Hartford county; M = Montgomery county; PG = Prince George’s county.

**Table 2.** Matrix of the four PM_2.5_ input surfaces (first column) used to form the baseline PMB and the four experimental aerosol optical depth (AOD)-PM_2.5_ fused surfaces (remaining columns).

PM_2.5_ Input Surfaces ^1^	Fused Surfaces ^2,3^				
	PMB	PMCQ	PMCKQ	PMC	PMCK
Monitor	X	X	X	X	X
CMAQ	X	X	X		
AOD-PM_2.5_		X		X	
AOD_K-_PM_2.5_			X		X

1 Input surfaces for different PM_2.5_ sources: Monitor = ambient PM_2.5_ air monitor measurements; CMAQ = Community Multiscale Air Quality PM_2.5_ model estimates; AOD-PM_2.5_ = AOD-PM_2.5_ with missing readings; AOD_K-_PM_2.5_ = Kriged AOD-PM_2.5_ without missing readings.

2 Hierarchical Bayesian Model (HBM) was used to form the five fused surfaces, including baseline PMB.

3 AOD-PM_2.5_ fused surfaces with (PMCQ, PMC) and without (PMCKQ, PMCK) missing readings.

**Table 3.** Three examples of bidirectional spatial lag grid case-crossover analyses, by ambient PM_2.5_ air monitor status: no monitors, monitors, and both monitor grid conditions combined.

Grid Monitor Examples ^1^	Spatial Lag Grid Analyses ^2^							
	0	1	2	3	4	01	24	04
No Monitors								
Rows (S to N)	**R_9_**	**R_9_**	**R_9_**	**R_9_**	**R_9_**	**R_9_**	**R_9_**	**R_9_**
Columns (W to E)	**C_7_**	**C_6_**	**C_5_**	**C_4_**	**C_3_**	**C_6–7_**	**C_3–5_**	**C_3–7_**
Monitors								
Rows (S to N)	**R_6_**	**R_6_**	**R_7_**	**R_7_**	**R_7_**	**R_6_**	**R_7_**	**R_6, 7_**
Columns (W to E)	**C_7_**	**C_6_**	**C_7_**	**C_6_**	**C_5_**	**C_6–7_**	**C_5–7_**	**C_6–7, 5–7_**
Both—All Grids								
Rows (S to N)	**R_4_**	**R_4_**	**R_4_**	**R_4_**	**R_4_**	**R_4_**	**R_4_**	**R_4_**
Columns (W to E)	**C_7_**	**C_6_**	**C_5_**	**C_4_**	**C_3_**	**C_7–6_**	**C_5–3_**	**C_7–3_**

1 Row (S to N) and column (W to E) grid coordinates for the Baltimore study area are shown here. Grids with air monitors are shown in red hue and in bold font, while grids without air monitors are displayed in black hue and in bold font. The sorting sequence was the column variable (1 to 9) first and the row variable (1 to 11) second.

2 Lag grid 0 is the index grid. Lag grid 1 refers to the grid that spatially preceded the index grid by 1 grid distance, 12 km. Lag grids 2 through 4 preceded the index grid by 2 to 4 grids, 24 km to 48 km, respectively. Lag grid 01 represents the mean of grid values 0 and 1. Lag grid 24 refers to the mean for grids 2–4. Lag grid 04 represents the mean for lag grids 0–4.

**Table 4.** Correlations between baseline PMB and the four AOD-PM_2.5_ fused surfaces by ambient PM_2.5_ air monitor status in the Baltimore study area.

Fused Surface	CMAQ Grid Ambient PM_2.5_ Air Monitor Status ^1,2^			
	Both (*n* = 8316)	Yes (*n* = 1260)	No (*n* = 7056)	∆%
PMC	0.676 (45.7) ^‡^	0.858 (73.6) ^‡^	0.642 (41.2) ^‡^	32.4
PMCK	0.553 (30.6) ^‡^	0.788 (62.1) ^‡^	0.515 (26.4) ^‡^	35.7
PMCQ	0.973 (94.7) ^‡^	0.987 (97.4) ^‡^	0.971 (94.3) ^‡^	3.1
PMCKQ	0.852 (72.6) ^‡^	0.928 (86.1) ^‡^	0.838 (70.2) ^‡^	15.9

1 Each cell contains correlation (*r^2%^*) values.

2 Significance identified with:

‡ = *p ≤* 0.01.

**Table 5.** Three-year mean concentration levels for the four AOD-PM_2.5_ and baseline PMB fused surfaces by air monitor grid status in the Baltimore study area.

Fused Surface	CMAQ Grid Ambient PM_2.5_ Air Monitor Status ^1,2^		
	Both	Yes	No
PMB	14.19 (14.13–14.26) ^†^	14.60 (14.44–14.76)	14.12 (14.05–14.19) ^†^,^‡^
PMC	13.66 (13.60–13.72) ^†^	13.90 (13.73–14.06) ^†^	13.62 (13.55–13.68) ^†^,^‡^
PMCK	14.38 (14.31–14.44)	14.27 (14.10–14.44) ^†^	14.39 (14.32–14.47)
PMCQ	13.79 (13.74–13.85) ^†^	14.28 (14.12–14.43) ^†^	13.71 (13.64–13.77) ^†^,^‡^
PMCKQ	13.91 (13.85–13.97) ^†^	14.24 (14.09–14.40) ^†^	13.85 (13.79–13.92) ^†^,^‡^

1 Each cell contains mean (95% CI) values in µg/m^3^.

2 Significant differences between means are based on the comparison mean exceeding the upper or lower 95% CI values of the control mean:

† = *p ≤* 0.05 (within each column);

‡ = *p ≤* 0.05 (between last two columns).

**Table 6.** Categorical analyses of fused surfaces, poverty percent, and population density by monitor status in the Baltimore study area.

Fused Surfaces and Demographic Categories ^1^	CMAQ Grid Ambient PM_2.5_ Air Monitor Status ^2–4^		
	Both	Yes	No
PMB—Below	38 (38.38) ^‡^	4 (26.67) ^†^	34 (40.48) ^‡^
Within	6 (6.06)	1 (6.67)	5 (5.95)
Above	55 (55.56)	10 (66.67/18.18)	45 (53.57/81.82) ^‡^
PMC—Below	52 (52.53) ^‡^	7 (46.67)	45 (53.57) ^‡^
Within	17 (17.17)	1 (6.67)	16 (19.05)
Above	30 (30.30)	7 (46.67/23.33)	23 (27.38/76.77) ^‡^
PMCK—Below	36 (36.36) ^‡^	8 (53.33)	28 (33.33) ^‡^
Within	18 (18.18)	2 (13.33)	16 (19.05)
Above	45 (45.45)	5 (33.33/11.11)	40 (47.62/88.89) ^‡^
PMCQ—Below	39 (39.39) ^‡^	3 (20.0) ^†^	36 (42.86) ^‡^
Within	7 (7.07)	2 (13.13)	5 (5.95)
Above	53 (53.54)	10 (66.67/18.87)	43 (51.19/81.13) ^‡^
PMCKQ—Below	38 (38.38) ^‡^	4 (26.67)	34 (40.48) ^‡^
Within	9 (9.09)	2 (13.33)	7 (8.33)
Above	52 (52.53)	9 (60.00/17.31)	43 (51.19/82.69) ^‡^
Poverty—Below	51 (75.00) ^‡^	7 (50.00)	44 (81.48) ^‡^
Within	2 (2.94)	0 (0.00)	2 (3.70)
Above	15 (22.06)	7 (50.00/46.67)	8 (14.81/53.33)
Population—Below	36 (52.94) ^‡^	3 (21.43) ^†^	33 (61.11) ^‡^
Within	2 (2.94)	0 (0.00)	2 (3.70)
Above	30 (44.12)	11 (78.57/36.67)	19 (35.19/63.33)

1 The Below, Within, and Above categories represent cutoffs for the 95% CI lower and upper limits for each fused surface concentration level and each demographic variable three-year mean values. Fused surface three-year means (95% CI) in Table 5 were used to determine cutoff values for the three-level categories.

2 These are the means (95% CIs) for the two demographic variables: poverty percent, 8.27 (95% CI, 7.84–8.69); population density (Log 10), 2.77 (95% CI, 2.75–2.78).

3 Statistical analyses were completed for the three-level categories (Below, Within, Above) in the three grid monitor conditions (columns 2–4), and for grids with and without air monitors in the Above category (rows; columns 3–4).

4 Significance is based on the chi-square test:

† = *p ≤* 0.05;

‡ = *p ≤* 0.01.

**Table 7.** Demographic attributes for respiratory–cardiovascular chronic disease cases and controls and patients’ race by monitor status in the Baltimore study area.

Variables	CMAQ Grid Ambient PM_2.5_ Air Monitor Status ^1,2^		
	Both	Yes	No
ED Asthma	47,256 (100.00)	20,815 (44.05)	26,441 (55.95)
Cases	11,723 (24.81)	5152 (10.90)	6571 (13.91)
Controls	35,533 (75.19)	15,663 (33.14)	19,870 (42.05)
Black ^‡^	22,696 (48.24)	11,844 (25.17)	10,852 (23.06)
Other	3060 (6.50)	785 (1.67)	2275 (4.84)
White	21,294 (45.26)	8082 (17.18)	13,212 (28.08)
IP Asthma	13,515 (100.00)	5672 (41.97)	7843 (58.03)
Cases	3376 (24.98)	1417 (10.48)	1959 (14.50)
Controls	10,139 (75.02)	4255 (31.48)	5884 (43.54)
Black ^‡^	4510 (33.43)	2358 (17.48)	2152 (15.95)
Other	669 (4.96)	179 (1.33)	490 (3.63)
White	8312 (61.61)	3119 (23.12)	5193 (38.49)
IP MI	19,021 (100.00)	7185 (37.42)	12,016 (62.58)
Cases	4790 (24.95)	1784 (9.29)	3006 (15.66)
Controls	14,411 (75.05)	5401 (28.13)	9010 (46.92)
Black ^‡^	2456 (13.28)	1183 (6.17)	1363 (7.11)
Other	848 (4.42)	180 (0.94)	668 (3.48)
White	15,780 (82.30)	5811 (30.31)	9969 (51.99)
IP HF	27,518 (100.0)	11,834 (43.00)	15,684 (57.00)
Cases	6826 (24.81)	2928 (10.64)	3898 (14.17)
Controls	20,692 (75.19)	8906 (32.36)	11,786 (42.83)
Black ^‡^	7029 (25.57)	3463 (12.60)	3566 (12.97)
Other	793 (2.88)	285 (1.04)	508 (1.85)
White	19,672 (71.55)	8078 (29.38)	11,594 (42.17)

1 Total grids with health data = 72: 15 with monitors, and 57 without monitors.

2 Number (%); Chi-Square,

‡ = *p ≤* 0.01.

**Table 8.** Lag grid analyses identified the size of homogeneous spatial areas (HOSAs), in multiples of one 12-km-wide Community Multiscale Air Quality (CMAQ) grid, when the AOD-PM_2.5_ fused surface ORs are greater than the baseline PMB ORs (Top), and the AOD–PM_2.5_ and baseline PMB fused surface ORs in grids without air monitors are compared to ORs in grids with air monitors (Bottom).

Grid Monitors ^1^	Respiratory-Cardiovascular Chronic Disease Groups			
	ED Asthma	IP Asthma	IP MI	IP HF
Both				
PMC	4 (0, 1, 01, 04)	3 (0, 1, 01)	3 (0, 1, 01)	3 (0, 1, 01)
PMCK	4 (0, 1, 01, 04)	3 (0, 1, 01)	3 (0, 1, 01)	4 (0, 1, 01, 04)
PMCQ	0	0	0	0
PMCKQ	4 (0, 1, 01, 04)	3 (0, 1, 01)	3, (0, 1, 01)	3 0, 1, 01)
Yes				
PMC	0	0	0	0
PMCK	2 (0, 1, 01)	0	0	0
PMCQ	0	0	0	0
PMCKQ	0	0	0	0
No				
PMC	4 (0, 1, 01, 04)	3 (0, 1, 01)	4 (0, 1, 01, 04)	4 (0, 1, 01, 04)
PMCK	4 (0, 1, 01, 04)	3 (0, 1, 01)	4 (0, 1, 01, 04)	4 (0, 1, 01, 04)
PMCQ	3 (0, 1, 01)	0	0	0
PMCKQ	4 (0, 1, 01, 04)	3 (0, 1, 01)	3 (0, 1, 01)	3 (0, 1, 01)
Monitor–No Monitor ^2^				
PMB	=	=	=	=
PMC	< (0, 1, 01)	< (1, 01)	< (0, 1, 01)	< (0,1, 01)
PMCK	< (0, 1, 01)	< (0, 01)	< (0, 1, 01)	< (0, 1, 01)
PMCQ	=	=	=	=
PMCKQ	=	=	=	=

1 Each AOD-PM_2.5_ fused surface OR is compared to the baseline PMB fused surface OR (95% CI). Only significant differences are shown, *p ≤* 0.05.

2 AOD-PM_2.5_ and baseline PMB fused surface ORs in grids without air monitors are compared to ORs (95% CIs) in grids with air monitors. Significant outcome at *p ≤* 0.05 is displayed with the symbol “<”. A non-significant outcome for *p* > 0.05 is shown with “=”.

**Table 9.** House heating fuel used in Baltimore study area in Community Multiscale Air Quality Grids with or without ambient air monitors ^1^.

Variables	Grids with Ambient Air Monitors ^3^			
	Yes		No	Both
Utility/Bottled Gas	56.4		35.2	52.9
Electricity	32.8		44.1	34.7
Fuel Oil/Kerosine	9.8		17.9	11.1
Other ^2^	0.7		2.5	1.0
No Fuel	0.3		0.3	0.3
Temperature °F ^4^		57.32	55.60	55.86
		(55.50–58.13)	(55.26–55.94)	(55.55–56.18)

1 Results from the 2005–2007 American Community Survey three-year estimates.

2 Other fuel: coal/coke, wood, solar.

3 Heating source cell values are percentages.

4 Three-year mean temperature values are for 2004–2006.

**Table 10.** Spatial autocorrelations based on Moran’s *I* for the five fused surfaces and ambient temperature by season in the Baltimore study area.

Surfaces	Seasons ^1,2^					
	Warm		Cold		Both	
	* I*	* Z*	* I*	* Z*	* I*	* Z*
PMB	0.0538	35.00 ^‡^	0.1690	109.40 ^‡^	0.0995	129.10 ^‡^
PMC	0.0370	24.10 ^‡^	0.0027	1.91	0.9902	12.14 ^‡^
PMCK	0.0268	17.49 ^‡^	* −*0.0014	* −*0.75	0.0045	6.01 ^‡^
PMCQ	0.0456	29.70 ^‡^	0.1040	67.50 ^‡^	0.0700	91.00 ^‡^
PMCKQ	0.0414	26.90 ^‡^	0.0389	25.40 ^‡^	0.0353	45.90 ^‡^
Temp °F	0.0167	10.96 ^‡^	0.0363	23.70 ^‡^	0.0232	30.20 ^‡^

1 Positive *Z* value for Moran’s *I* represents positive autocorrelation, while negative *Z* value indicates negative autocorrelation, when *p ≤* 0.05.

2 *Z p* value:

‡ = *p ≤* 0.01.

## Data Availability

The Maryland Health Services Cost Review Commission provided one year’s access to confidential hospital data after payment of the data usage fee. It is not possible to share the electronic patient records since the electronic patient records contain information that could be used to identify the names and addresses of patients in the electronic ED and IP files.

## Abbreviations

∆%: Percent difference
∆OR%: No monitor–monitor OR percent
r^2%^: Square of correlation coefficient percent
AOD: Aerosol optical depth
CI: 95% confidence interval
CLR: Conditional logistic regression
CMAQ: Community Multiscale Air Quality
ED: Emergency department
HBM: Hierarchical Bayesian Model
HF: Heart failure
HOSA: Homogeneous spatial area
ICD-9-CM: International Classification of Diseases, Ninth Revision, Clinical Modification
IP: Inpatient hospitalization
MI: Myocardial infarction
OR: Odds ratio
PHREG: Proportional hazards regression
PM_0.1_: Ultrafine particulate matter
PM_2.5_: Fine particulate matter
PMB: PM_2.5_ baseline model (monitor PM_2.5_ and CMAQ PM_2.5_)
PMC: AOD PM_2.5_ model (monitor PM_2.5_ and AOD PM_2.5_)
PMCK: AOD PM_2.5_ Kriged model (monitor PM_2.5_ and AOD PM_2.5_ Kriged)
PMCKQ: AOD PM_2.5_ Kriged and CMAQ PM_2.5_ model (monitor PM_2.5_ and AOD PM_2.5_ Kriged and CMAQ PM_2.5_)
PMCQ: AOD PM_2.5_ and CMAQ PM_2.5_ model (monitor PM_2.5_ and AOD PM_2.5_ and CMAQ PM_2.5_)
SAS: Statistical analysis system
ZCTA: ZIP code tabulation area
ZIP Code: Zone improvement plan
