# Supplementary material for: New Homogeneous Spatial Areas Identified Using Case-Crossover Spatial Lag Grid Differences between Aerosol Optical Depth-PM2.5 and Respiratory-Cardiovascular Emergency Department Visits and Hospitalizations
Source: Atmosphere (Basel). Author manuscript; Available in PMC 2023 Apr 30. (PMC9393882; doi:10.3390/atmos13050719)
Supplement: Supplement1 — Figure S1: ED asthma ORs and 95% CIs for the four AOD-PM2.5 and baseline PMB fused surfaces during the warm and cold seasons at lag grids 0 (a), 1 (b), 01 (c), and 04 (d). Figure S2: IP asthma ORs and 95% CIs for the four AOD-PM2.5 and baseline PMB fused surfaces during the warm and cold seasons at lag grids 0 (a), 1 (b), 01 (c), and 04 (d). Figure S3: IP MI ORs and 95% CIs for the four AOD-PM2.5 and baseline PMB fused surfaces during the warm and cold seasons at lag grids 0 (a), 1 (b), 01 (c), and 04 (d). Figure S4: IP HF ORs and 95% CIs for the four AOD-PM2.5 and baseline PMB fused surfaces during the warm and cold seasons at lag grids 0 (a), 1 (b), 01 (c), and 04 (d). Table S1: No monitor–monitor ∆OR% for the four AOD-PM2.5 and baseline PMB fused surfaces and the four respiratory-cardiovascular ED visits and IP hospitalizations lag grid and lag day analyses. Table S2: Warm-cold season ∆OR% for the four AOD-PM2.5 and baseline PMB fused surfaces and four respiratory-cardiovascular ED visits and inpatient hospitalizations lag grid and lag day analyses. [file NIHMS1809138-supplement-Supplement1.pdf]

Supplementary File

Manuscript title: New Homogeneous Spatial Areas Identified Using Case-Crossover Spatial Lag Grid Differences between Aerosol Optical Depth-$PM_{2.5}$ and Respiratory-Cardiovascular Emergency Department Visits and Inpatient Hospitalizations

Journal name: *Atmosphere*

Author names: John T. Braggio, Eric S. Hall, Stephanie A. Weber, and Amy K. Huff

Corresponding author: John T. Braggio, Maryland Department of Health, Baltimore, MD, 21201, USA; Mt. Diablo Analytical Solutions and Reporting Institute, LLC (Diablo Analytical Institute, DAI), 3474 Tice Creek Drive, Unit 7, Walnut Creek, CA 94595, USA. Email; johntbs@msn.com; Cell: (+1) 510-520-5149 (permanent address)

Four Figures and Two Tables

Figure S1: ED asthma ORs (95% CIs) for the four AOD-$PM_{2.5}$ and baseline PMB fused surfaces during the warm and cold seasons at lag grids 0 (a), 1 (b), 01 (c), and 04 (d).

Figure S2: IP asthma ORs (95% CIs) for the four AOD-$PM_{2.5}$ and baseline PMB fused surfaces during the warm and cold seasons at lag grids 0 (a), 1 (b), 01 (c), and 04, (d).

Figure S3: IP MI ORs (95% CIs) for the four AOD-$PM_{2.5}$ and baseline PMB fused surfaces during the warm and cold seasons at lag grids 0 (a), 1 (b), 01 (c), and 04 (d).

Figure S4: IP HF ORs (95% CIs) for the four AOD-$PM_{2.5}$ and baseline PMB fused surfaces during the warm and cold seasons at lag grids 0 (a), 1 (b), 01 (c), and 04 (d).

Table S1: No monitor - Monitor OR Difference Percent (ΔOR%) for the Four AOD-$PM_{2.5}$ and Baseline PMB Fused Surfaces and the Four Respiratory-Cardiovascular ED Visits and IP Hospitalizations Spatial Lag Grid and Temporal Lag Day Analyses in the Baltimore Study Area.

Table S2: Warm - Cold Season OR Difference Percent (ΔOR%) for the Four AOD-$PM_{2.5}$ and Baseline PMB Fused Surfaces and the Four Respiratory-Cardiovascular ED Visits and IP Hospitalizations Spatial Lag Grid and Temporal Lag Day Analyses in the Baltimore Study Area.

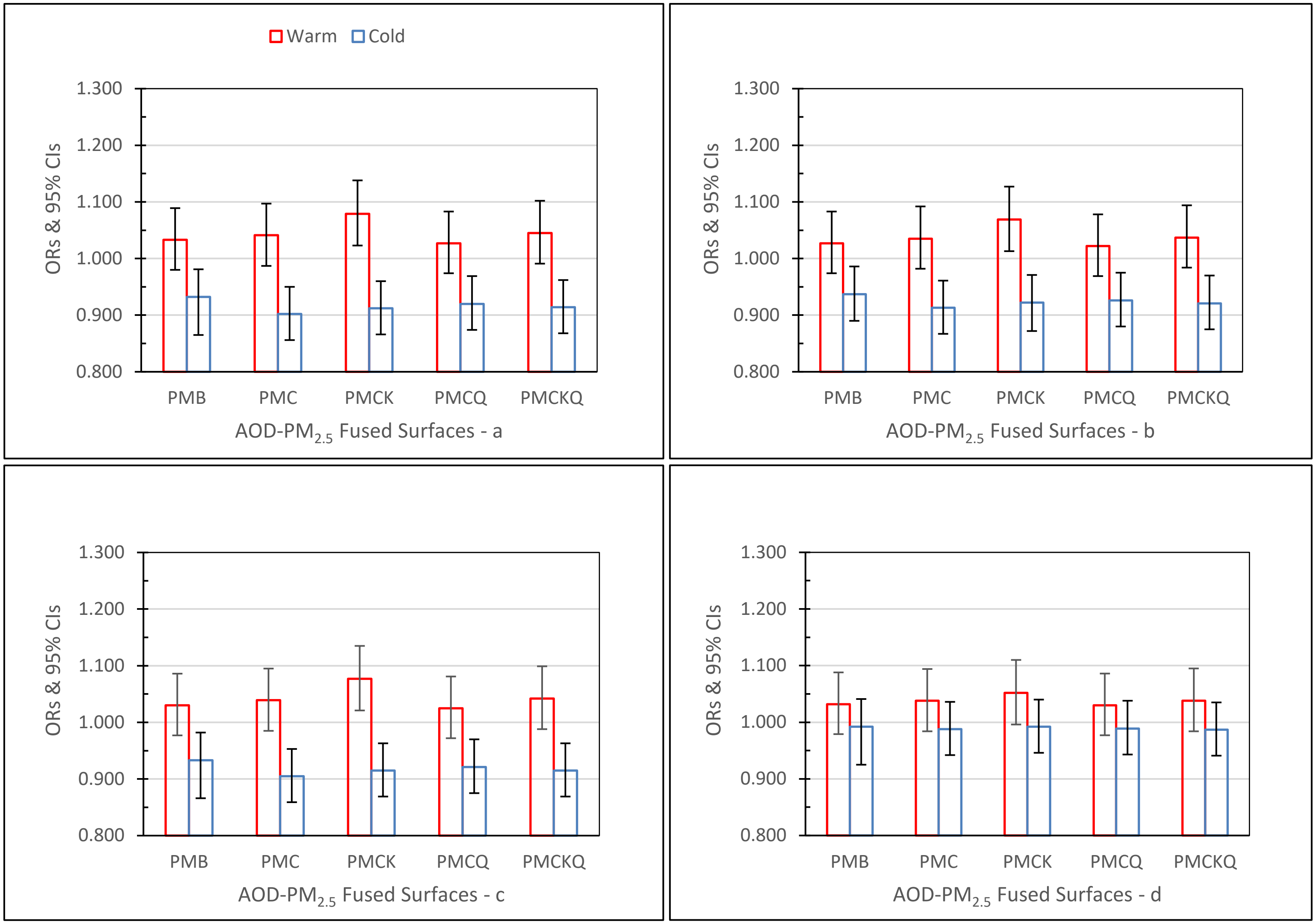


Figure S1: ED asthma ORs (95% CIs) for the four AOD-$PM_{2.5}$ and baseline PMB fused surfaces during the warm and cold seasons at lag grids 0 (a), 1 (b), 01 (c), and 04 (d).

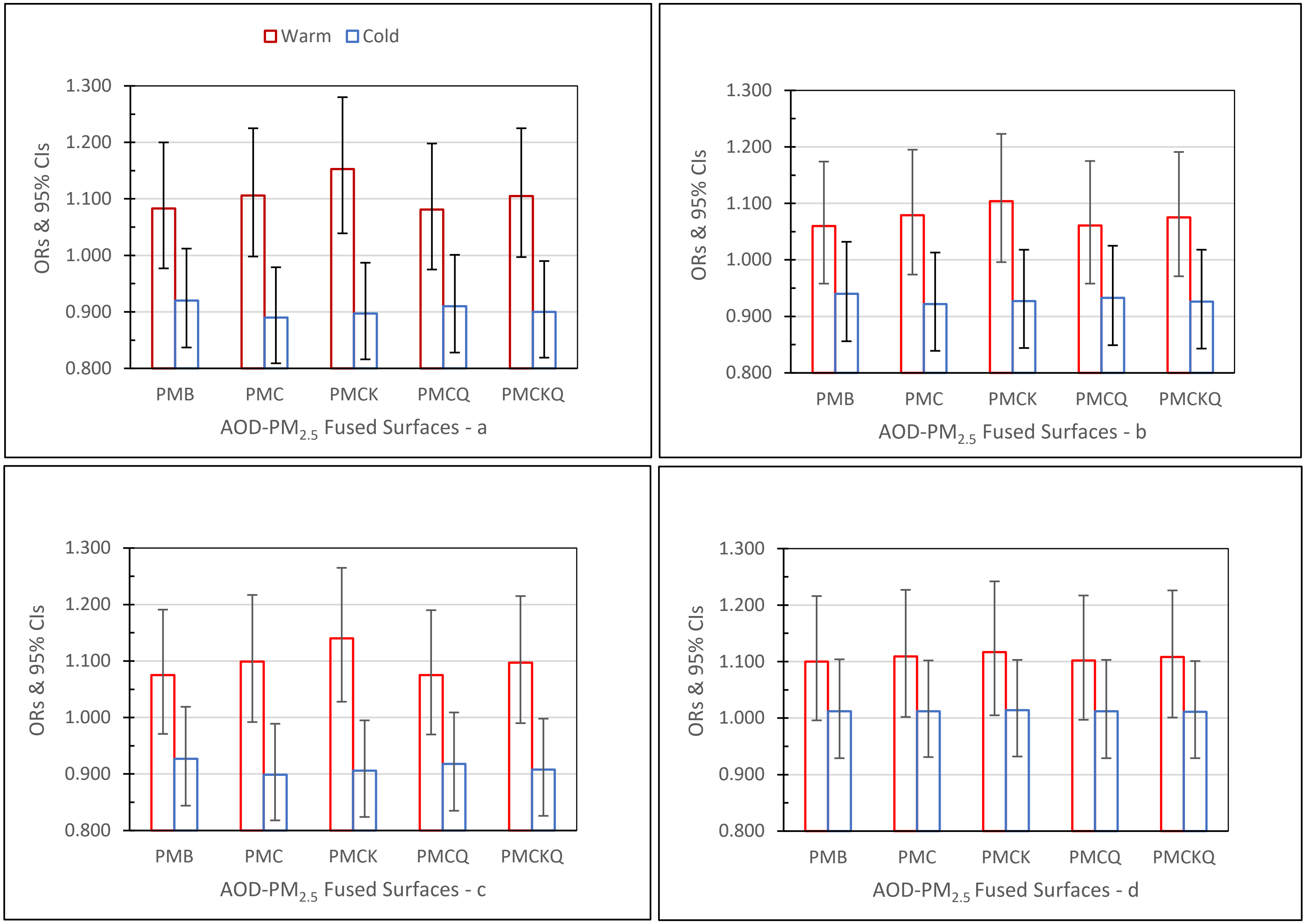


Figure S2: IP asthma ORs (95% CIs) for the four AOD-$PM_{2.5}$ and baseline PMB fused surfaces during the warm and cold seasons at lag grids 0 (a), 1 (b), 01, (c), and 04 (d).

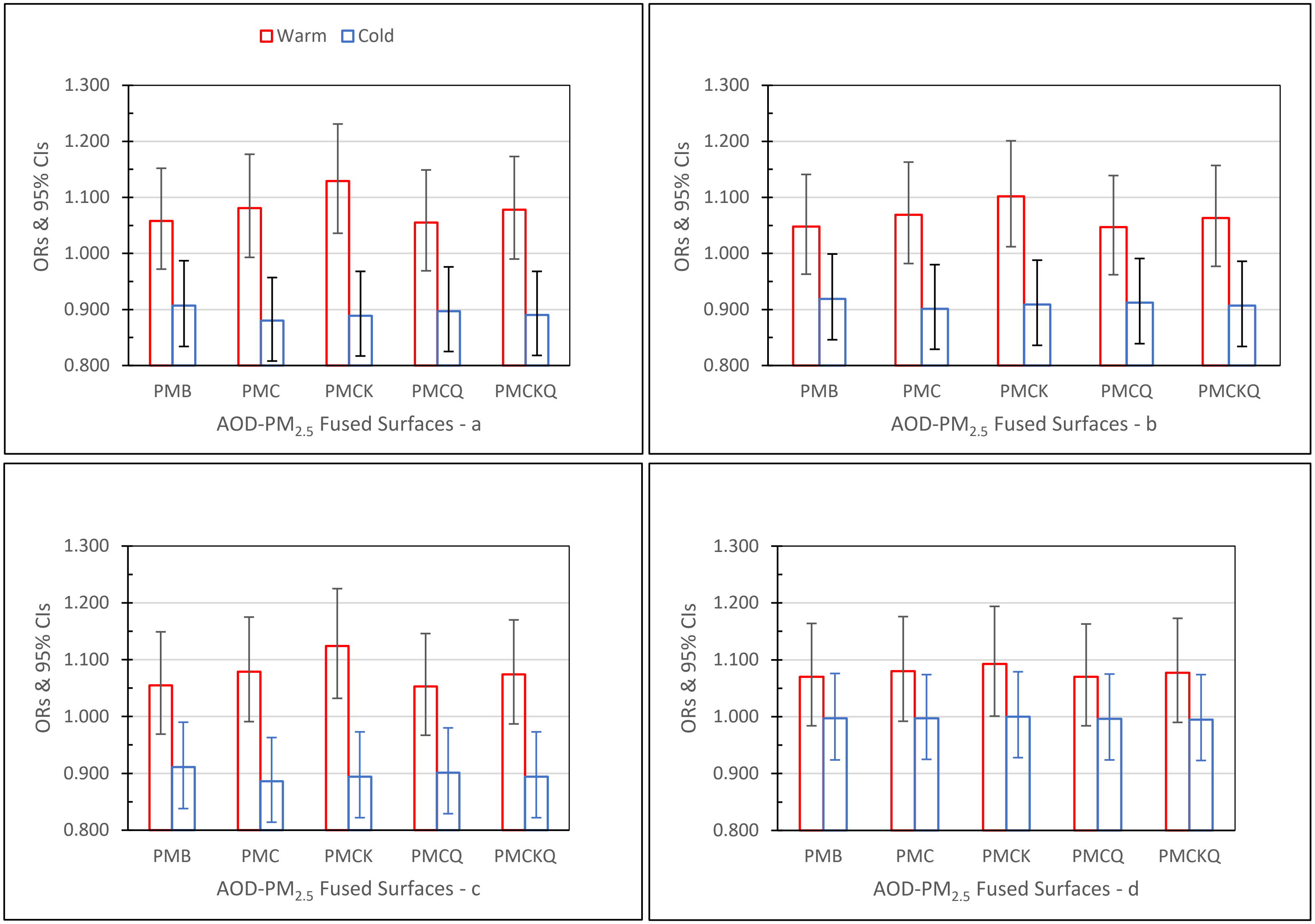


Figure S3: IP MI ORs (95% CIs) for the four AOD-$PM_{2.5}$ and baseline PMB fused surfaces during the warm and cold seasons at lag grids 0 (a), 1 (b), 01 (c), and 04 (d).

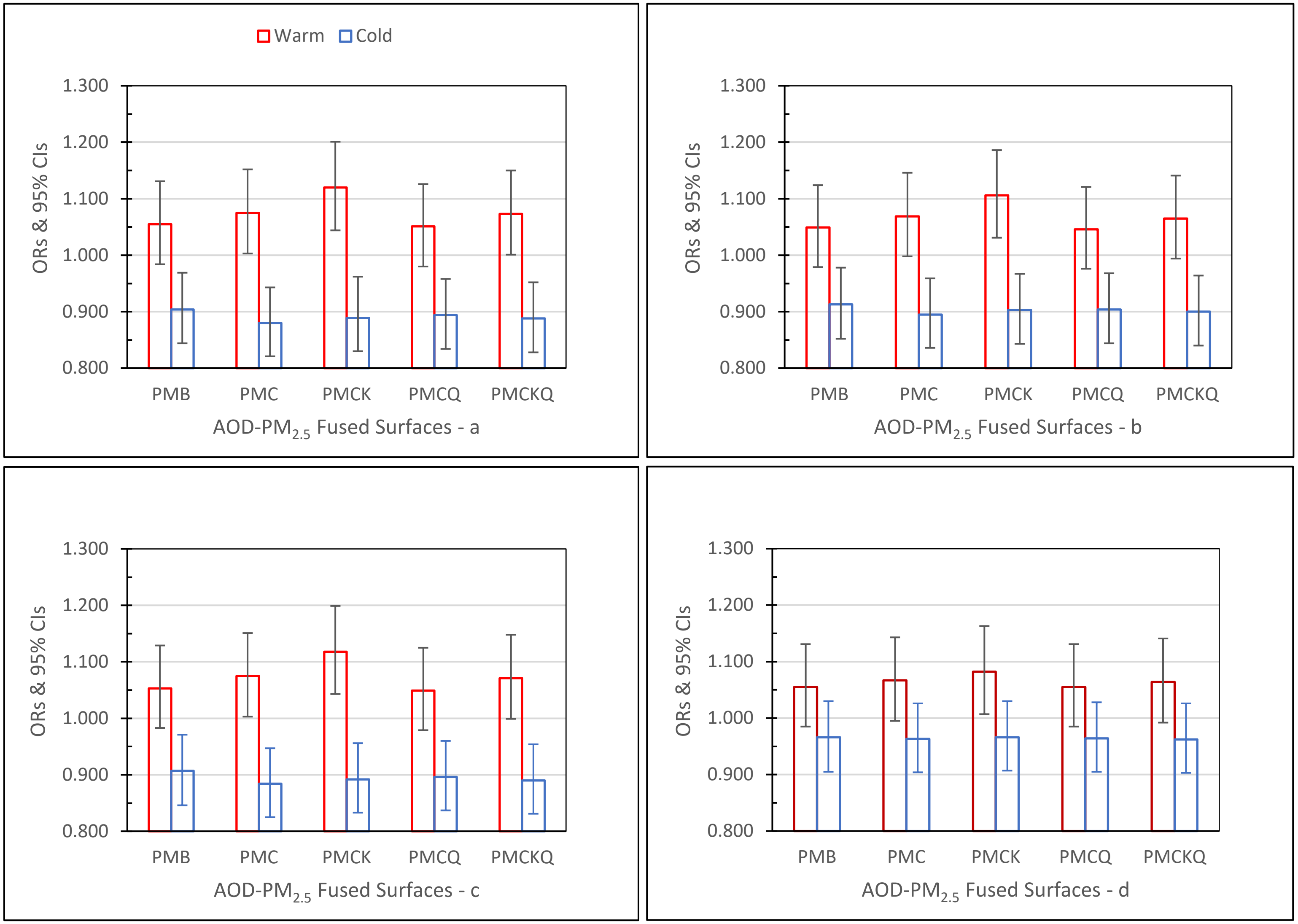


Figure S4: IP HF ORs (95% CIs) for the four AOD-$PM_{2.5}$ and baseline PMB fused surfaces during the warm and cold seasons at lag grids 0 (a), 1 (b), 01 (c), and 04 (d).

Table S1: No Monitor - Monitor OR Difference Percent (ΔOR%) for the Four AOD-$PM_{2.5}$ and Baseline PMB Fused Surfaces and the Four Respiratory-Cardiovascular ED Visits and IP Hospitalizations Spatial Lag Grid and Temporal Lag Day Analyses in the Baltimore Study Area.

| Case-Crossover Lag Grid or Lag Day Analyses[1-4] | | | | | | | | | |
|---|---|---|---|---|---|---|---|---|---|
| Lag Value | Fused Surfaces | Grids | | | | Days | | | |
| | | ED AS | IP AS | IP MI | IP HF | ED AS | IP AS | IP MI | IP HF |
| 0 | PMB | -0.7†,‡ | -0.9†,‡ | -0.7†,‡ | -0.6†,‡ | -0.7†,‡ | -0.9†,‡ | -0.7†,‡ | -0.6†,‡ |
| | PMC | 2.0†,† | 1.7†,‡ | 2.2†,‡ | 2.0†,‡ | 2.0†,‡ | 1.7†,‡ | 2.2†,‡ | 2.0†,‡ |
| | PMCK | 2.3†,‡ | 2.5†,‡ | 2.5†,‡ | 2.3†,‡ | 2.3†,‡ | 2.5†,‡ | 2.5†,‡ | 2.3†,‡ |
| | PMCQ | 0.0†,‡ | -0.4†,‡ | 0.0†,‡ | -0.1†,‡ | 0.0†,‡ | -0.4†,‡ | 0.0†,‡ | -0.1†,‡ |
| | PMCKQ | 0.7†,‡ | 0.5†,‡ | 0.7†,‡ | 0.7†,+ | 0.7†,‡ | 0.5†,‡ | 0.7†,‡ | 0.7†,‡ |
| 1 | PMB | -0.5†,‡ | -0.6†,‡ | -0.5†,‡ | -0.5†,‡ | -0.7†,‡ | -1.0†,‡ | -0.7†,‡ | -0.6†,‡ |
| | PMC | 1.9†,‡ | 1.1†,‡ | 1.8†,‡ | 1.8†,‡ | 2.1†,‡ | 1.6†,‡ | 2.1†,‡ | 1.9†,‡ |
| | PMCK | 2.1†,‡ | 1.6†,‡ | 2.0†,‡ | 2.1†,‡ | 2.2†,‡ | 2.4†,‡ | 2.4†,‡ | 2.3†,‡ |
| | PMCQ | 0.0†,‡ | -0.2†,‡ | 0.0†,‡ | 0.1†,‡ | 0.0†,‡ | -0.4†,‡ | 0.0†,‡ | -0.1†,‡ |
| | PMCKQ | 0.7†,‡ | 0.3†,‡ | 0.6†,‡ | 0.7†,‡ | 0.6†,‡ | 0.4*,‡ | 0.6†,‡ | 0.7†,‡ |
| 01 | PMB | -0.6†,‡ | -0.8†,‡ | -0.5†,‡ | -0.5†,‡ | -0.9†,‡ | -1.4†,‡ | -1.2†,‡ | -1.1†,‡ |
| | PMC | 2.2†,‡ | 1.7†,‡ | 2.3†,‡ | 2.1†,‡ | 4.5†,‡ | 4.3†,‡ | 5.0†,‡ | 4.3†,‡ |
| | PMCK | 2.5†,‡ | 2.6†,‡ | 2.7†,‡ | 2.6†,‡ | 4.7†,‡ | 5.7†,‡ | 5.6†,‡ | 4.8†,‡ |
| | PMCQ | 0.0†,‡ | -0.3†,‡ | 0.1†,‡ | 0.1†,‡ | 0.2†,‡ | -0.4†,‡ | 0.0†,‡ | 0.1†,‡ |
| | PMCKQ | 0.9†,‡ | 0.7†,‡ | 0.8†,‡ | 0.9†,‡ | 1.4†,‡ | 1.0†,‡ | 1.1†,‡ | 1.2†,‡ |
| 04 | PMB | -0.6,‡ | -0.5 | -0.6‡ | -0.3†,‡ | | | | |
| | PMC | 0.1†,‡ | 0.0† | 0.3†,‡ | 0.5†,‡ | | | | |
| | PMCK | 0.2†,‡ | 0.3† | 0.5†,‡ | 0.5†,‡ | | | | |
| | PMCQ | -0.4†,‡ | -0.4 | -0.4†,‡ | -0.2†,‡ | | | | |
| | PMCKQ | -0.3†,‡ | -0.2 | -0.2†,‡ | -0.1†,‡ | | | | |

[1]Each cell includes no monitor - monitor ΔOR% values. [2]Significance of ORs for monitor grid conditions (grids without [no] or with [yes] air monitors), $p \leq 0.05$: † = no, ‡ = yes. [3]Red hue indicates higher lag day value than lag grid value, while blue hue represents lower lag day value than lag grid value. [4]Underlined cell value identifies a difference between the significance of the outcome for a lag grid OR and a lag day OR.

Table S2: Warm - Cold Season OR Difference Percent (ΔOR%) for the four AOD-$PM_{2.5}$ and Baseline PMB Fused Surfaces and the Four Respiratory-Cardiovascular ED Visits and IP Hospitalizations Spatial Lag Grid and Temporal Lag Day Analyses in the Baltimore Study Area.

| Case-Crossover Lag Grid or Lag Day Analyses[1-4] | | | | | | | | | |
|---|---|---|---|---|---|---|---|---|---|
| Lag Value | Fused Surfaces | Grids | | | | Days | | | |
| | | ED AS | IP AS | IP MI | IP HF | ED AS | IP AS | IP MI | IP HF |
| 0 | PMB | 10.8‡ | 17.7 | 16.6‡ | 16.7‡ | 10.8‡ | 17.7 | 16.6‡ | 16.7‡ |
| | PMC | 15.4‡ | 24.3‡ | 22.8‡ | 22.2†,‡ | 15.4‡ | 24.3‡ | 22.8‡ | 22.2†,‡ |
| | PMCK | 18.3†,‡ | 28.5†,‡ | 27.0†,‡ | 26.0†,‡ | 18.3†,‡ | 28.5†,‡ | 27.0†,‡ | 26.0†,‡ |
| | PMCQ | 11.6‡ | 18.8 | 17.6‡ | 17.6‡ | 11.6‡ | 18.8 | 17.6‡ | 17.6‡ |
| | PMCKQ | 14.3‡ | 22.8‡ | 21.1‡ | 20.8†,‡ | 14.3‡ | 22.8‡ | 21.1‡ | 20.8†,‡ |
| 1 | PMB | 9.6‡ | 12.8 | 14.0‡ | 14.9‡ | 10.6‡ | 16.1 | 16.2‡ | 16.3‡ |
| | PMC | 13.4‡ | 17.0 | 18.6‡ | 19.4‡ | 14.7‡ | 21.9‡ | 21.9‡ | 21.5†,‡ |
| | PMCK | 15.9†,‡ | 19.1 | 21.2†,‡ | 22.5†,‡ | 17.5†,‡ | 25.4†,‡ | 25.6†,‡ | 24.9†,‡ |
| | PMCQ | 10.4‡ | 13.7 | 14.8‡ | 15.7‡ | 11.4‡ | 17.0 | 17.0‡ | 17.2‡ |
| | PMCKQ | 12.6‡ | 16.1 | 17.2‡ | 18.3‡ | 13.9‡ | 20.7‡ | 20.4‡ | 20.3‡ |
| 01 | PMB | 10.4‡ | 16.0 | 15.8‡ | 16.1‡ | 17.4‡ | 26.8‡ | 26.2‡ | 25.1‡ |
| | PMC | 14.8‡ | 22.2‡ | 21.8‡ | 21.6†,‡ | 24.6‡ | 38.1†,‡ | 36.7†,‡ | 33.9†,‡ |
| | PMCK | 17.7†,‡ | 25.8†,‡ | 25.7†,‡ | 25.3†,‡ | 28.4†,‡ | 45.2†,‡ | 44.1†,‡ | 40.5†,‡ |
| | PMCQ | 11.3‡ | 17.1‡ | 16.9‡ | 17.1‡ | 18.7‡ | 28.6‡ | 27.8+ | 26.6‡ |
| | PMCKQ | 11.3‡ | 20.8‡ | 20.1‡ | 20.3‡ | 23.1‡ | 33.1†,‡ | 33.9†,‡ | 32.2†,‡ |
| 04 | PMB | 4.0 | 8.7 | 7.3 | 9.2 | | | | |
| | PMC | 5.1 | 9.6 | 8.3 | 10.8 | | | | |
| | PMCK | 6.0 | 10.2† | 9.3† | 12.0† | | | | |
| | PMCQ | 4.1 | 8.9 | 7.4 | 9.4 | | | | |
| | PMCKQ | 5.2 | 9.6 | 8.2 | 10.6 | | | | |

[1]Each cell includes warm - cold season ΔOR% values. [2]Significance of warm-cold season ORs, $p \leq 0.05$: † = warm; ‡ = cold. [3]Red hue indicates a higher lag day OR value than a lag grid OR value. [4]Underlined cell value identified a difference in the significance of the outcome for a lag grid OR and a lag day OR.
